# Bioinformatic analyses to uncover genes involved in trehalose metabolism in the polyploid sugarcane

**DOI:** 10.1038/s41598-022-11508-x

**Published:** 2022-05-07

**Authors:** Lauana Pereira de Oliveira, Bruno Viana Navarro, João Pedro de Jesus Pereira, Adriana Rios Lopes, Marina C. M. Martins, Diego Mauricio Riaño-Pachón, Marcos Silveira Buckeridge

**Affiliations:** 1 Laboratório de Fisiologia Ecológica de Plantas, Departamento de Botânica, Instituto de Biociências, Universidade de São Paulo, São Paulo, Brazil; 2 Laboratório de Bioquímica, Instituto Butantan, São Paulo, Brazil; 3 Laboratório de Biologia Computacional, Centro de Energia Nuclear na Agricultura, Evolutiva e de Sistemas, Universidade de São Paulo, São Paulo, Brazil; 4 Instituto Nacional de Ciência e Tecnologia do Bioetanol, São Paulo, Brazil

**Keywords:** Biotechnology, Genetics, Molecular biology, Transcription

## Abstract

Trehalose-6-phosphate (T6P) is an intermediate of trehalose biosynthesis that plays an essential role in plant metabolism and development. Here, we comprehensively analyzed sequences from enzymes of trehalose metabolism in sugarcane, one of the main crops used for bioenergy production. We identified protein domains, phylogeny, and in silico expression levels for all classes of enzymes. However, post-translational modifications and residues involved in catalysis and substrate binding were analyzed only in trehalose-6-phosphate synthase (TPS) sequences. We retrieved 71 putative full-length TPS, 93 trehalose-6-phosphate phosphatase (TPP), and 3 trehalase (TRE) of sugarcane, showing all their conserved domains, respectively. Putative TPS (Classes I and II) and TPP sugarcane sequences were categorized into well-known groups reported in the literature. We measured the expression levels of the sequences from one sugarcane leaf transcriptomic dataset. Furthermore, TPS Class I has specific *N*-glycosylation sites inserted in conserved motifs and carries catalytic and binding residues in its TPS domain. Some of these residues are mutated in TPS Class II members, which implies loss of enzyme activity. Our approach retrieved many homo(eo)logous sequences for genes involved in trehalose metabolism, paving the way to discover the role of T6P signaling in sugarcane.

## Introduction

Sugars, mainly sucrose, lie at the heart of plant metabolism. During photosynthesis, plants synthesize sucrose that is transiently stored in vacuoles, used for cellular activities, and exported from source to sink tissues to sustain metabolism and growth^1,2^. Simultaneously, starch accumulates in the leaf chloroplasts as short-term storage degraded during the night to meet the continuous carbon demand. Thus, plants regulate their sugar levels in temporal and spatial scales.

**Figure 1:**
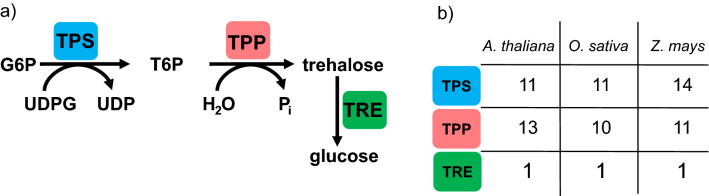
Plant trehalose metabolism. (**a**) Enzymes involved in trehalose synthesis and degradation. (**b**) The number of genes encoding trehalose-6-phosphate synthase (TPS), trehalose-6-phosphate phosphatase (TPP), and trehalase (TRE) in *Arabidopsis thaliana*, *Oryza sativa*, and *Zea mays*. Trehalose-6-phosphate (T6P) is a central sugar sensor in plant metabolism. Its biosynthesis occurs from UDP-glucose (UDPG) and glucose-6-phosphate (G6P) by TPS activity. Subsequently, the T6P is converted to trehalose by TPP. Trehalose is hydrolyzed into two molecules of glucose by TRE. Multigenic families encode TPS and TPP.

The appropriate balance among carbon assimilation, partitioning, and use is critical for plant development, survival, and reproductive success. Sugars function as substrates for growth and affect sugar-sensing systems that regulate how, when, and where sugars are utilized. Although both abundance and depletion of sugars significantly affect gene expression^3,4^, resolving the mechanisms and physiological significance of sugar signaling in plants has proved to be challenging. This depends on multiple pathways that respond to different sorts of sugar, which interact with each other and in conjunction with additional nutrients (e.g., nitrogen and phosphorus) and the environmental and phytohormone responses^5–9^. As a signaling molecule, trehalose-6-phosphate (T6P), the intermediate of trehalose biosynthesis, is a sucrose-specific signal in plants and has a far-reaching influence on metabolism, growth, and development^10^. Therefore, T6P is a potential target for improving model and crop plants. Despite multiple trehalose biosynthetic routes, the only one found in plants involves the enzymes trehalose-6-phosphate synthase (TPS) and trehalose-6-phosphate phosphatase (TPP)^11^. TPS catalyzes the transfer of glucose from UDP-glucose to glucose-6-phosphate, producing T6P and uridine diphosphate. TPP dephosphorylates T6P to form trehalose and inorganic phosphate. Trehalose is a nonreducing disaccharide involved in osmoregulation and stress protection and its breakdown in several organisms occurs via the hydrolytic enzyme trehalase (TRE), resulting in the formation of two glucose molecules (Fig. 1).

TPS and TPP enzymes are encoded by multigenic families divided into distinct subfamilies based on their sequence similarity to the homologs *Saccharomyces cerevisiae* TPS (*ScTps1*) or TPP (*ScTps2*)^12–14^. *Arabidopsis thaliana*, *Oryza sativa*, and *Zea mays* TPS genes are divided into Class I (*AtTPS1–4*, *OsTPS1*, and *ZmTPSI.1.1-1.2*) and Class II (*AtTPS5-11*, *OsTPS2-11*, and *ZmTPSII.2.1-5.4*), both having a glycosyltransferase family 20 domain (TPS domain) and a trehalose-phosphatase domain (TPP domain)^15,16^. Class I’s TPS domain is closely related to the *ScTps1* and *Escherichia coli* TPS (*otsA*), while Class II encodes proteins with higher similarity to *ScTps2*. Except for *AtTPS3*, only the Class I isoforms have catalytic activity confirmed by the complementation of yeast mutants^17–20^. Several Class II TPS genes respond to sugar availability^21–24^ and hormones^18,25,26^, besides displaying diurnal cycles of expression ^27^. However, the function of most Class II genes remains enigmatic, although it has been suggested that they play regulatory roles. Arabidopsis has ten TPP genes (*AtTPPA*-*AtTPPJ*) containing a TPP domain, three HAD motifs also found in Class II TPS proteins, and a highly variable N-terminal region of unknown function^14^. All *AtTPP* genes originated from whole-genome duplication and their encoded proteins are catalytically functional^28^. Poaceae species present similar quantities of *TPP* genes: 10 in rice and 11 in maize^15,16^. Different from *TPS* and *TPP*, *AtTRE* is encoded by a single gene. Based on the distribution of TPS, TPP, and TRE genes, trehalose metabolism appears to be universal in the plant kingdom and has ancient origins^12–14^.

T6P levels are positively correlated with sucrose in several tissues, developmental stages, and species^29,30^. T6P was proposed as a signal of sucrose availability that also exerts a negative feedback regulation on sucrose levels to maintain them within a proper range according to the cellular metabolic status and the plant developmental stage^31,32^. However, this function is unclear in species such as sugarcane (*Saccharum* spp), accumulating a large amount of sucrose in its stems. This species is an important crop worldwide used as feedstock for sugar and bioethanol production. Brazil was the first country to introduce bioethanol as an efficient renewable fuel for transportation and stands out as one of the largest bioethanol exporters^33^. Improving sugar production per unit area and/or sucrose concentration in the stems has been an important goal of breeding programs^34,35^. Nevertheless, gains in sucrose content in commercial sugarcane varieties are about 1.0–1.5% per year and are believed to be near their limit^36–38^.

The sugarcane genome is complex, interspecific, polyploid, and displays extensive aneuploidy^39^, but research in sugarcane genomics has advanced^40–51^. One monoploid mosaic reference genome for the sugarcane hybrid R570 was released^52^ along with a high-quality chromosome level genome for *S. spontaneum*, one of the parentals of sugarcane hybrids^53^. Nevertheless, the incomplete coverage of the whole genome still hampers sugarcane biotechnological improvement. We identified the sequences and evolutionary relationships among *A. thaliana* and Panicoideae members of gene families encoding enzymes involved in trehalose metabolism. We combined search of orthologous genes in Viridiplantae, phylogenetic analysis, identification of functional protein domains and residues involved in catalysis and binding. These analyses were further combined with three-dimensional structure prediction, post-translational modifications, and in silico expression profiles. This approach should establish a foundation for further functional studies to uncover the physiological roles of T6P signaling in sugarcane. Such knowledge will help decipher the regulation of carbon partitioning and allocation, essential for the more efficient conversion of sugarcane biomass into bioproducts.

## Results

### Identification of sugarcane sequences involved in trehalose metabolism

Protein sequences of TPS, TPP, and TRE from *A. thaliana*, *Z. mays*, and *O. sativa* were used as queries to identify the orthologous groups (OGs) that they have been assigned to in the EggNOG database^54^. These OGs made it possible to retrieve homologous sequences from other Viridiplantae species (Supplementary Table S1). Subsequently, publicly available transcriptomic and genomic datasets from sugarcane (Table 1) were annotated with EggNOG.

**Table 1:** Publicly available datasets used to identify genes encoding trehalose metabolizing enzymes in sugarcane.

Dataset/Abbreviation	Type of dataset	Variety—Species	Tissue	Sequencing technology	BioProject
SCA5^55^	Expressed Sequence Tag (EST)	> 3 varieties (Including SP80-3280)	Different tissues	ABI sequencer	sucest-fun.org*
SCA3^56^	Transcriptomic	SP80-3280	Leaf	Illumina Hi-Seq2500	PRJNA244522
SCA4^49^	Genomic	SP80-3280	Leaf	Illumina HiSeq2000	PRJNA272769
SAC2_1 and SAC2_2^47^	Transcriptomic	10 varieties	Internode	Illumina HiSeq4000	PRJNA356226
	Transcriptomic	22 varieties	Leaf, internode and root	PacBio	
SCA1_1 and SCA1_2^52^	Transcriptomic	R570	leaves, roots, and stems	Illumina Hi-Seq2500	ERZ654945**
	Genomics	R570, *S. spontaneum* and *S. officinarum*		BAC, PacBio and Illumina WGS	
SSP^53^	Genomics	*S. spontaneum*	Leaf	BAC, Illumina HiSeq 2500, PacBio and Illumina HiSeq X Ten platform	PRJNA483885

*Data available in http://sucest-fun.org/.

**Data deposited in the EMBL-European Bioinformatics Institute.

**Figure 2:**
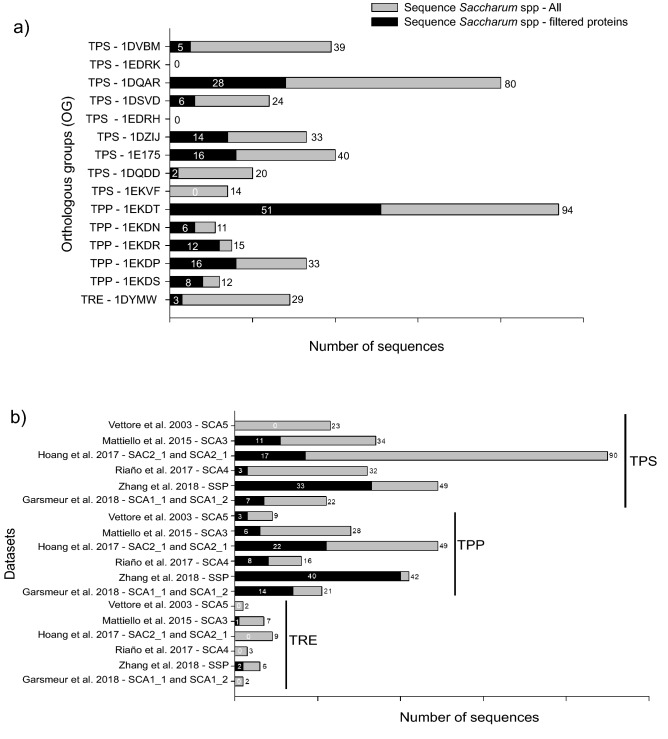
The number of sequences related to the trehalose metabolism pathway in sugarcane. (**a**) Dispersion of total (grey) and filtered (black) sequences associated with orthologous groups (OG) from TPS, TPP, and TRE. The sequences related to each OG are described in Supplementary Table S1. (**b**) Dispersion of total (grey) and filtered (black) sequences among sugarcane publicly available datasets. The datasets (sequence IDs) analyzed were described in Table 1: SCA5^55^; SCA3^56^; SCA4^49^; SAC2_1 and SCA2_1^47^; SCA1_1 and SCA1_2^52^; SSP^53^.

The OGs obtained from model species and sugarcane were joined, recovering 15 OGs: nine for TPS, five for TPP, and one for TRE (Supplementary Table S1). The search on the sugarcane databases retrieved 444 sequences from all homo(eo)logous targets (Table 2). This is conceivable because the datasets used represent distinct sugarcane cultivars or genotypes, including the *S. spontaneum* genome (Fig. 2b). Three OGs were excluded from further analyses: OG 1EKVF presented WD40 domains, which did not correspond to TPS proteins; OG 1EDRK and 1EDRH (Supplementary Table S1) contained only Arabidopsis sequences. These findings left us with 12 OGs and 430 sequences. To further assess the phylogenetic relationships among *Saccharum* spp homo(eo)logous sequences, 12 amino acid-based phylogenies were constructed, one for each OG (Supplementary Table S1 and Supplementary Fig. S1: a-l).

**Table 2:** Number of putative trehalose pathway protein sequences found in the species under study, using the EggNOG database. The defined OGs for sequences from model species and sugarcane were joined, recovering 15 OGs.

Species	TPS class I		TPS class II	TPP	TRE
	B1	B2	A1-A5	(A1, A2, A3, B1 and B2)	
*Arabidopsis thaliana*	4	5	19	22	1
*Oryza sativa*	2	0	21	20	1
*Triticum aestivum*	18	0	24	37	3
*Setaria* spp	3	0	20	27	2
*Sorghum bicolor*	2	0	25	39	2
*Miscanthus sinensis*	5	0	24	31	9
*Zea mays*	55	0	120	26	6
*Saccharum* spp	39	0	211	165	29

Numbers of putative protein sequences of trehalose-6-phosphate synthase (TPS)—Class I (catalytic) and II (regulatory), trehalose-6-phosphate phosphatase (TPP), and trehalase (TRE) in different plant species. The clade distribution follows the nomenclature established by^15,16^.

### The protein domains are conserved in a few retrieved sugarcane sequences

The presence of conserved protein domains in sequences of sugarcane, *Sorghum bicolor*, and *A. thaliana* was verified with HMMER scan^57^. Both glycosyltransferase family 20 (TPS domain—PF00982) and trehalose-phosphatase (TPP domain—PF02358) domains were found for all Class I and II TPS sequences. For TPP family and TRE single domains, a TPP and a trehalase were obtained, respectively (Supplementary Fig. S1: a-l). Only sugarcane sequences having ≥ 80% of their respective model domains (71 TPS, 93 TPP, and three TRE) (Fig. 2a and b) were considered filtered and had their proteins shown in the phylogenetic trees (Supplementary Table S1 and Supplementary Fig. S1: a-l). Most of the retrieved sequences from the sugarcane databases used in this study do not harbor their respective protein domains or have them incomplete and thus are most likely transcript or protein fragments. These results reinforce the importance of using different datasets when working with polyploid species that still lack well-annotated genomes.

### Classification of TPS and TPP sequences from sugarcane

**Figure 3:**
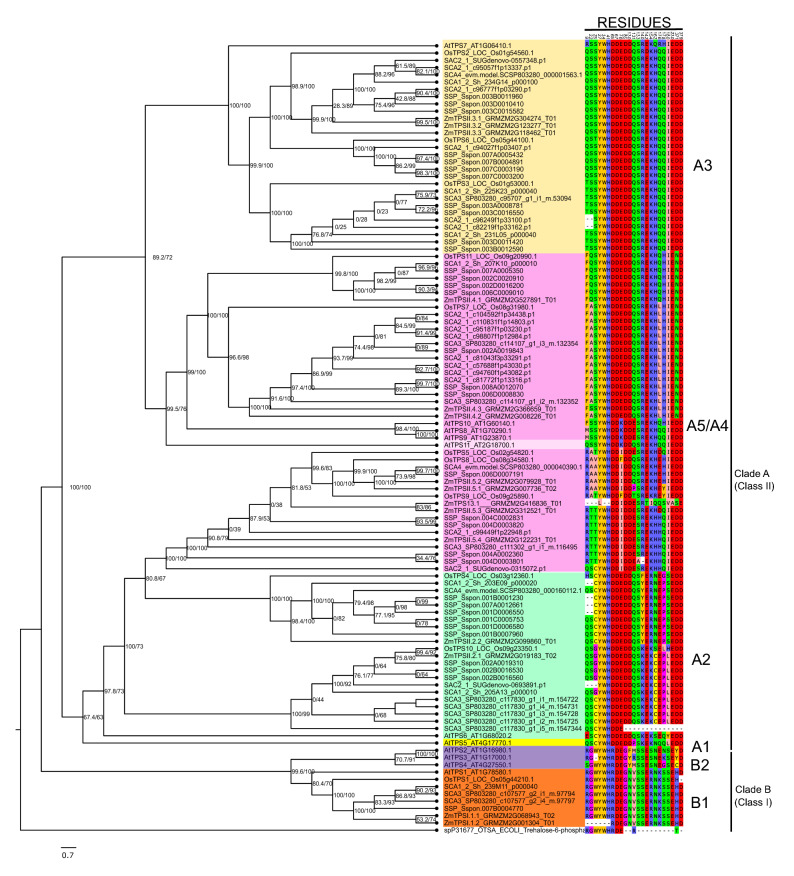
Phylogenetic tree of trehalose-6-phosphate synthase (TPS) from sugarcane, *Zea mays*, *Oryza sativa*, and *Arabidopsis thaliana*. Filtered sugarcane sequences were used to construct a phylogenetic tree based on previous protein sequences obtained from^15,16^. The tree was built with IQ-TREE^59^ using automatic evolutionary model selection, branch support values are shown as SH-like aLRT (%)^60^ and ultrafast bootstrap (UFboot) (%)^61^. Branches with SH-like aLRT > 80% and UFboot > 95% are confident. Previous established clades are shown, although they are not always supported by the topology^15,16^. Residues related with the division of subclades of TPS Class I and II are highlighted. Databases and accession numbers are listed in Supplementary Table S1.

To classify the filtered sugarcane sequences based on previously established clades^15,16^, the TPS and TPP amino acid-based phylogenetic trees were rebuilt. TPS Class I (clade B) and Class II (clade A) could be distinguished (Fig. 3). Clade B is divided into two (B1 and B2). As observed in other studies, gene sequences belonging to subclade B2 were unique to *A. thaliana*^58^. Subclades A1 to A5 can also be identified as some topological disagreements arose, although not well endorsed by the computed low branch support values. Most of the sequences already classified in previous studies (*A. thaliana*, maize, and rice)^15,16^ remained in their clades, but AtTPS11 was not well resolved, making it difficult to distinguish between A4 and A5 subclades.

**Figure 4:**
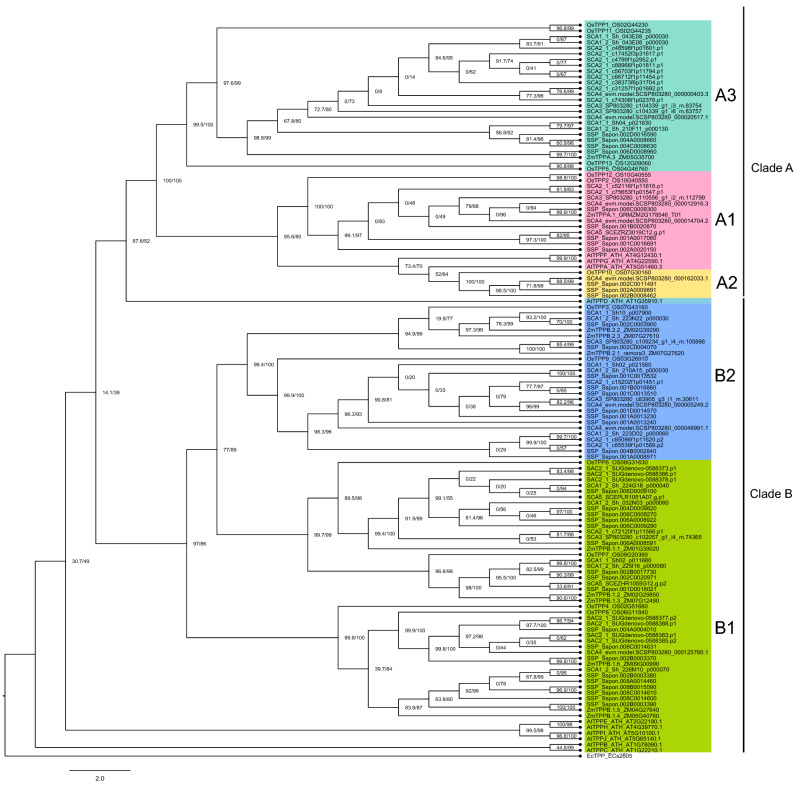
Phylogenetic tree of trehalose-6-phosphate phosphatase (TPP) from sugarcane, *Zea mays*, *Oryza sativa*, and *Arabidopsis thaliana*. Filtered sugarcane sequences were used to construct a phylogenetic tree based on previous protein sequences obtained from^16^. The tree was built with IQ-TREE^59^ using automatic evolutionary model selection, branch support values are shown as SH-like aLRT (%)^60^ and ultrafast bootstrap (UFboot) (%)^61^. Branches with SH-like aLRT > 80% and UFboot > 95% are confident. Previous established clades are shown, although they are not always supported by the topology^16^. Databases and accession numbers are listed in Supplementary Table S1.

Similarly, as for the TPS family, our analysis of TPPs recovered most of the previously identified clades but with some topological disagreements. The TPP family is displayed in two clades (A and B) divided into three (A1 to A3) and two (B1 and B2) subclades. In this study, subclades A2 and A3 were unique to monocots, and AtTPPD was grouped in clade B2 instead of B1 (Fig. 4). Besides, the classification in OGs by EggNOG does not necessarily reflect the different subclades in the phylogenetic analyses for both TPS and TPP.

### Identity analysis of TPS, TPP, and TRE in sugarcane

**Figure 5:**
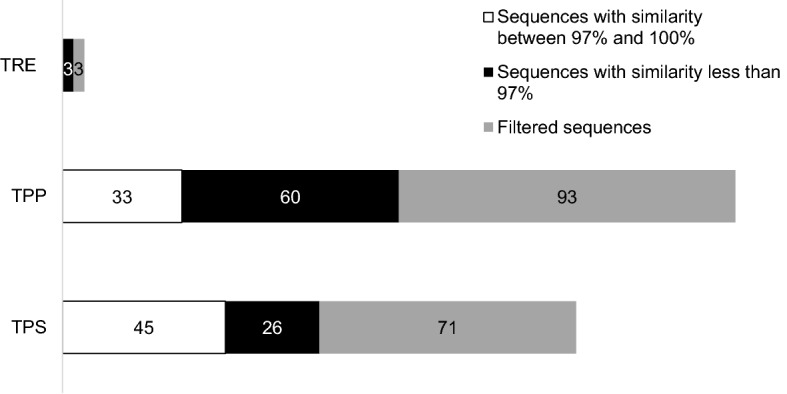
Identity of global pairwise alignment for multiple filtered sequences. Pairwise sequence alignments between all pairs of sequences were computed with the software needle from the EMBOSS v6.0.0. suite, using default settings.

Because most of the trehalose metabolizing enzymes have isoforms with high similarity in some cases^62^ and we retrieved the corresponding sugarcane sequences from different datasets (Fig. 2b), the TPS, TPP, and TRE filtered sequences were submitted to identity analysis. A global pairwise alignment for multiple sequences was performed for each of the three targets. From all filtered sequences, 14 TPS and 13 TPP were identified as redundant sequences with 100% of identity (Fig. 5). Likewise, 26 TPS, 60 TPP, and three TRE sequences showed an identity of ≤ 97% (Fig. 5 and Supplementary Table S2). Our findings indicate that different datasets (from different cultivars/different sequencing approaches) could recover some identical sequences. Additionally, highly similar sequences could represent alternative splice variants or recent paralogs.

### In silico expression in sugarcane leaves

**Figure 6:**
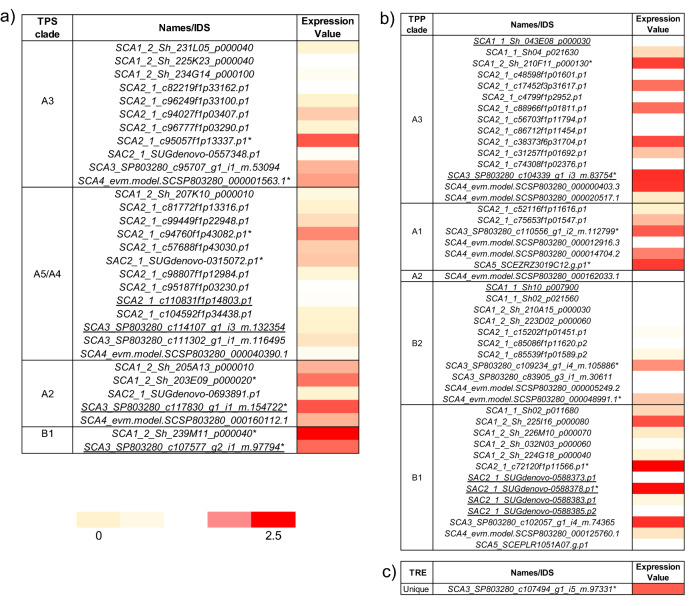
Expression values of trehalose-6-phosphate synthase (*TPS*) (**a**), trehalose-6-phosphate phosphatase (*TPP*) (**b**), and trehalase (*TRE*) (**c**). The heatmaps show the transformed TPM [log_10_(TPM + 1)], using a subset of the transcriptomics dataset from^56^. The sugarcane cultivar SP80-3280 was grown in a greenhouse for 60 days and gene expression in the portion of the leaf with the highest photosynthetic activity (middle part of the leaf + 1) was assessed. Red and yellow indicate high and low expression values, respectively. Each clade has the sequences obtained from the selected datasets (Table 1), which were related to the expression values accessed in^56^. Sequence IDs followed by an asterisk indicate sequences with higher expression value in each clade and underlined sequence IDs specify clustered sequences*.*

To provide evidence of how *TPS*, *TPP*, and *TRE* are expressed in sugarcane leaves (var. SP80-3280), we analyzed a published RNA-seq data^56^. Except for *TRE*, we recovered the expression levels (Transcripts Per Million—TPM) of sequences from most databases used in this analysis (please see materials and methods for details), including 31 *TPS* and 46 *TPP* from distinct clades (Fig. 6a–c). TPP clade A3 contained the largest number (15) of expressed sequences. For TPS clades with regulatory functions (A2, A3, and, A4/A5) *SCA3_SP803280_c117830_g1_i1_m.154722*, *SCA2_1_c95057f1p13337.p1*, and *SCA2_1_c94760f1p43082.p1* had higher expression values. Interestingly, the sequence with the highest expression levels (2.5 -fold higher) (*SCA1_2_Sh_239M11_p000040*) among all *TPS* belongs to clade B1 (Class I), characterized by sequences with catalytic function (Fig. 6a). The *TPP* transcripts with the maximum expression values were *SCA5_SCEZRZ3019C12.g.p1*, *SCA3_SP803280_c104339_g1_i3_m.83754*, and *SCA2_1_c72120f1p11566.p1*. These sequences belong to clades A1, A3, and B1, respectively (Fig. 6b). *TPP* sequences in clades A2 and B2 were not expressed. *TRE* showed a unique sequence with a low expression value (1.5) compared with the above-mentioned *TPS* and *TPP* sequences (Fig. 6c).

### Class I TPS harbors specific predicted *N*-glycosylation sites in conserved motifs

**Figure 7:**
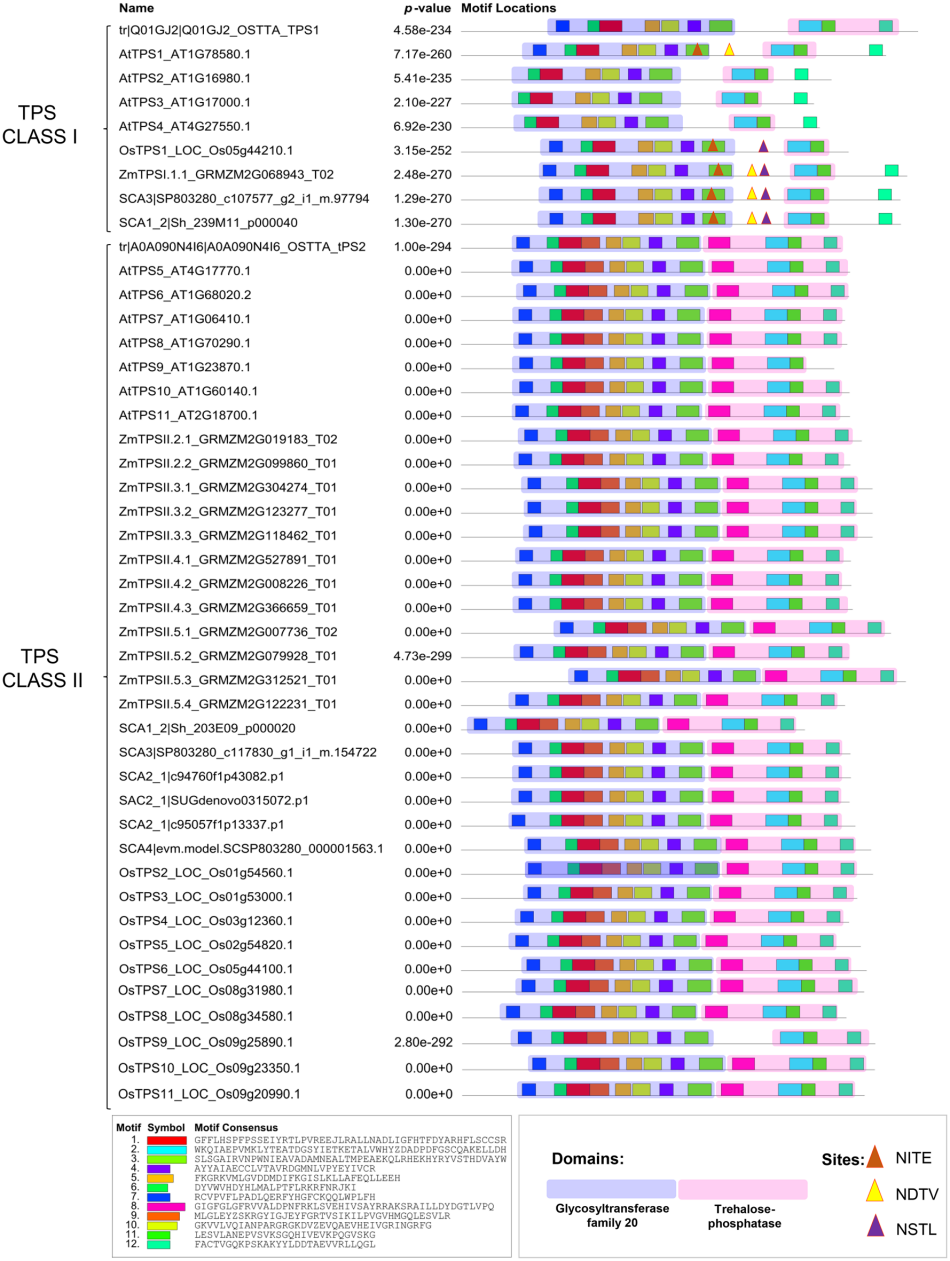
Conserved motifs of trehalose-6-phosphate synthase (TPS) protein sequences showing *N*-glycosylation sites. Motif analysis was performed using the MEME online program. A total of 12 putative conserved motifs of sugarcane TPS Class I and II proteins was identified and classified as overlapped with the glycosyltransferase (transparent blue) or trehalose-phosphatase (transparent red) domains. Prediction of *N*-glycosylation sites were investigated for TPS sequences of *O. tauri*, *A. thaliana*, *Z. mays*, *O. sativa*, and *Saccharum* spp by NetNGlyc 1.0. Three *N*-glycosylation sites in Class I TPS are demonstrated (NITE, NDTV, and NSTL). All *N*-glycosylation sites are listed in Supplementary Table S3.

We evaluated the putative glycosylation sites (Supplementary Table S3) and conserved motifs (Fig. 7) among filtered TPS Class I and II sequences. Sequences of *Ostreococcus tauri* that have two TPS, one Class I and one Class II enzyme^14^, were included in this analysis.

Many sequences (73.8%) harbors predicted *N*-glycosylation sites (Asn), in which 17 and 41 were exclusive to Class I and II, respectively, whereas three appeared in both classes (Supplementary Table S3). NDTV, NITE, and NSTL sites were detected in Class I sequences for most species, including *A. thaliana*, *Z.mays*, *O. sativa*, and sugarcane. NITE is localized in motif 3 and overlapped with the TPS domain in AtTPS1 and monocot species (Fig. 7). Otherwise, the NDTV and NSTL sites were predicted to be located between the two domains (Fig. 7), with NDTV present in the same sequences highlighted above (except rice) and NSTL only in monocot species (Supplementary Table S3). For OtTPS1 (*O. tauri*), AtTPS1 (Arabidopsis), OsTPS1 (rice), ZmTPSI.1.1 (maize), and two sugarcane sequences (SCA3_SP803280_c107577_g2_i1_m.97794 and SCA1_2_Sh_239M11_p000040) of the Class I, the putative motifs 1, 3, 4, 5, 6, 7, and 10 were conserved and together constitute the TPS domain. Interestingly, in other isoforms of *A. thaliana* TPS Class I (AtTPS2, AtTPS3, and AtTPS4) motif 7 was not present. The putative motifs 2 and 11 constitute the TPP domain in all Class I TPS except for *O. tauri*. All sequences of TPS Class II contained the same motifs present in the TPS and TPP domains. Additionally, motif 9 from the TPS domain and motif 8 from the TPP domain were not presented in TPS Class I.

### TPS Class I and II catalytic and binding residues show mutations at TPS and TPP domains

Sugarcane and *A. thaliana* protein sequences that had ≥ 80% similarity with the respective model domains were used to construct three-dimensional (3D) structures. Crystallized proteins of *Aspergillus fumigatus* (Model 5hvm.1.A—TPS), *Mycobacterium tuberculosis* (Model 5gvx.1.A—TPP), and *E. coli* (Model 2jjb.1.A—TRE) were defined as templates in SWISS-MODEL^63^. The templates had at least 30% sequence identity for TPS, 31% for TPP, and 35% for TRE (Supplementary Table S1 and Supplementary Fig. S2). When the protein folding was analyzed, TPS proteins shared similar structures independently from their catalytic (Class I) or regulatory (Class II) function based on the phylogenetic classification (data not shown). Alternatively, TPP 3D structures seem different when comparing the monocot (sugarcane) and eudicot (*A. thaliana*). The latter is closest to the template (Supplementary Fig. S2).

To distinguish sugarcane catalytic TPS Class I from the regulatory Class II, we aimed at identifying all residues involved in catalysis and binding from each domain and their putative mutations. For that, the two sugarcane sequences with the highest expression levels from each clade (Fig. 6a), as well as those from *A. thaliana*, *O. sativa*, *Z. mays*, and *O. tauri* (Supplementary Fig. S3), were aligned to the TPS from *E. coli* (OtsA)^64^, *Candida albicans* (Tps1)^65^, and *C. albicans* TPP (Tps2)^66^. This alignment (Supplementary Fig. S3) showed that for TPS Class I, all species listed above presented catalytic and binding residues already described for OtsA at the TPS domain. The TPS Class II presented mutations at the TPS domain, implying in loss of enzyme activity (Table 3). Moreover, the TPP domain of all Class I and Class II sugarcane TPS have remarkable similarities with the TPP domain of *C. albicans* Tps2^66^, displaying mutations that allow the differentiation between catalytic and regulatory classes (Supplementary Fig. S3 and Table 3).

**Table 3:** Identification of catalytic, binding residues, and substitution at TPS Class I and II from *Saccharum* spp.

	TPS class I		TPS class II	
	Catalytic	Binding	Catalytic	Binding
TPS domain	R_9_-R	E_171_-E	**R-Q**	E-E
	G_22_-G	R_370_-R	**G-S**	R-R
	W_41_-W		**W–C**	
	Y_76_-Y		Y-Y	
	W_85_-W		W-W	
	D_130_-D		D-D	
	H_154_-H		H–H	
	R_162_-R		**R-D**	
	D_371_-D		D-D	
	E_379_-E		E-E	
TPP domain	**D**_**25**_**-G**	**D**_**27**_**-N**	D-D	D-D
	S_65_-S	**P**_**32**_**-E**	S–S	**P-/**
	K_188_-K	V_34_-V	K-K	**V-Q**
	**D**_**230**_**-H**	**R**_**67**_**-S**	D-D	**R-K**
	D_234_-D	E_131_-E	D-D	E-E
		**K**_**133**_**-R**		K-K
		**H**_**140**_**-N**		**H-C**
		**R**_**142**_**-K**		**R-E**
		**K**_**176**_**-S**		**K-P**
		**N**_**178**_**-S**		**N-S**
		E_180_-E		E-E

TPS domain catalytic and binding residues were identified based on *E. coli* TPS^64,66^. TPP domain catalytic and binding residues were identified based on *Candida albicans* TPP^66^. TPS Class I and TPS II from *Saccharum* spp. residues are based on the analysis of two sequences more expressed SCA1_2Sh_23M11_p000040 (catalytic) and SCA3_SP803280_c117830_g1_il_m.154722 (regulatory). Bolded residues indicate residue replacement and / indicates a deletion. The numeration in residues are based on^66^, for example, mutations at X25Z. Other residues replacements are described in Supplementary Figure S3 at the phylogenetic tree analysis (Fig. 3).

**Figure 8:**
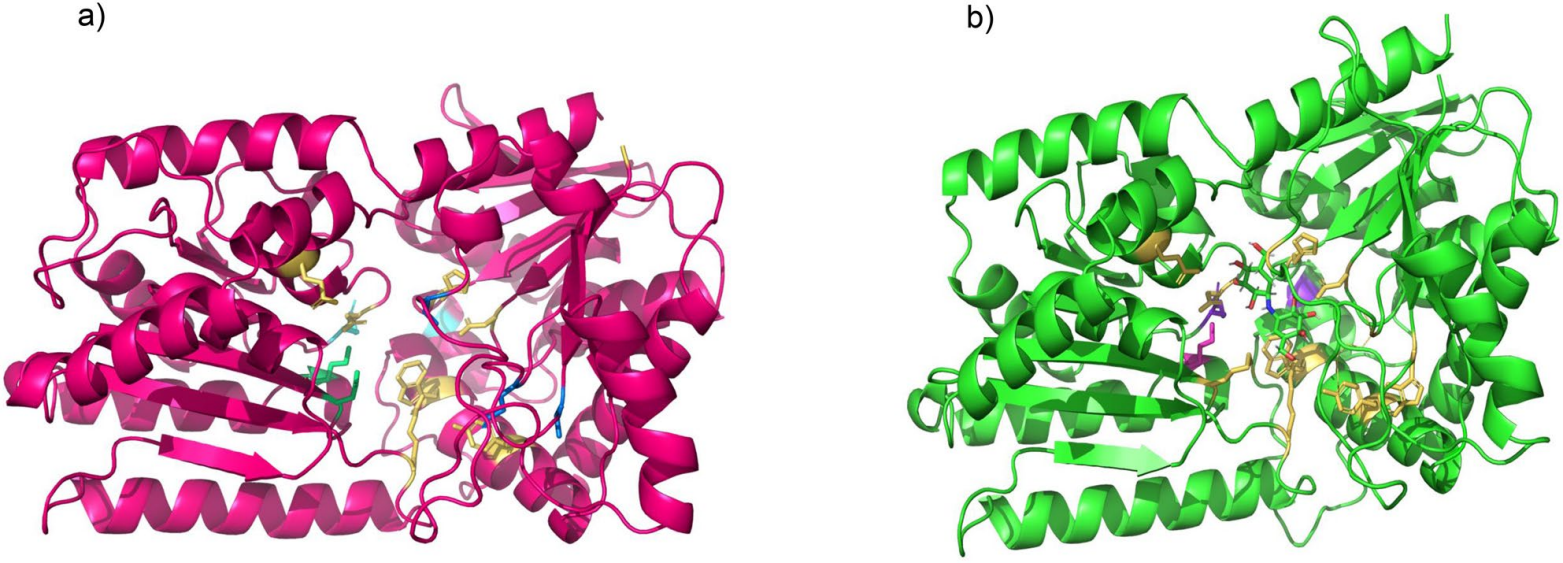
Three-dimensional (3D) structures of trehalose-6-phosphate synthase (TPS) Class I and II. 3D template structures of TPS from *A. fumigatus* (ID model 5hvm.1.A) were used as models to predict the sugarcane TPS Class I and II structure. (**a**) Sugarcane TPS Class I structure (SCA1_2_Sh239M11_p000040): yellow marked residues comprise the catalytic residues, purple-blue residues are involved in oligomer interaction, and light magenta highlights the K residue at the R/K pair. This analysis indicates that this enzyme is active and contains all catalytic residues. (**b**) Sugarcane TPS Class II structure (SCA3_SP803280_c117830_g1_i1_m154722): marine blue indicates modified catalytic residues; yellow-orange catalytic conserved residues, ciane residues are involved in oligomer interaction, and lime green highlights K residue at the R/K pair.

The deduced proteins from the most highly expressed sugarcane TPS Class I and II enzyme-coding transcripts (SCA1_2Sh_23M11_p000040 and SCA3_SP803280_c117830_g1_il_m.154722, respectively) were submitted to 3D structure analysis using SWISS-MODEL (Fig. 8). For sugarcane TPS Class I, two distinct templates, 5hut (Qmean of − 1.7562, sequence ID 50.1%) and 5hvm (Qmean of -1.6795, sequence ID 50.75%), appeared as best hits, and therefore 5hvm was chosen. The same template was used for the sugarcane TPS Class II.

Both sugarcane TPS Class I (Fig. 8a) and II (Fig. 8b) displayed similar folding structures (Supplementary Fig. S2). However, sugarcane TPS Class I presented mutations at D25G (the change of D in *C. albicans* to G in sugarcane at position 25 of the *C. albicans*) catalytic residue of TPP domain and at the binding residues D27N, R67S, K133R, H140N, R142K, K176S, and N178S. TPS Class II displayed the replacement of residues involved in binding at the TPP domain (Table 3), mainly at R67K. Besides, the division of TPS Class II subclades is related to specific amino acid replacements involved in substrate binding or catalysis residues on both domains (Fig. 8). For instance, residue R9 at the TPS domain, in which Q/T replaces at A3 and A2 subclade, and F/M at subclade A5 (Fig. 3).

## Discussion

Sugarcane plays a key role in the Brazilian bioenergy sector regarding economic and societal aspects^67^, such as environmental sustainability^68^. Gains in sugarcane yield have the potential to increase not only bioethanol production, an effective alternative to mitigate CO_2_ emissions and climate change, but also other bioproducts^33^. However, some efforts remain necessary to achieve essential improvements in productivity, for instance, dealing with the complexity of its genome.

Due to its high ploidy levels, presence of aneuploidy, high rates of polymorphism, and repeat content, the sugarcane genome is still a challenge for genome sequencing, contributing to the lack of information about molecular function and structure^52^. However, recent advances in sequencing technologies and computational strategies for genome assembly are opening the way to deciphering the sugarcane genome^49,52,53^. Combining different sequencing strategies to mine datasets makes it possible to retrieve more accurate information about homo(eo)logous sequences. Therefore, the present work resorted to bioinformatics methods to identify the sugarcane trehalose pathway-related targets, accessing distinct sugarcane datasets^47,49,52,53,55,56^. Altogether, 430 sequences related to the trehalose pathway were classified into 12 OGs from the EggNOG database. Only 36% (167 sequences) displayed all the predicted domains with high similarity (≥ 80%) to the established domain templates.

Identification of trehalose pathway-related target sequences in sugarcane is of particular interest to understand the mechanisms involved in sucrose accumulation since T6P is a specific sucrose sensor^31,32^. Information about trehalose metabolism in sugarcane is limited to the characterization of the TRE enzyme^69–71^ and transcriptomic studies that have identified changes in the expression of putative genes encoding TPS and TPP^56,72–76^. These findings are consistent with the hypothesis that T6P could be a master key sensor in this species. Most of these studies have inferred the role of trehalose metabolism on abiotic stress tolerance and regulation of photosynthesis. However, the precise identification of the isoforms was unfeasible. Trehalose levels in sugarcane culms were five orders of magnitude lower than sucrose, ranging from less than 0.3 to 3.9 nmol g^-1^FW, although these sugars did not correlate linearly^77^. In contrast^78^, showed that trehalose positively correlated with sucrose. Thus, the correlation between these two disaccharides remains unclear for sugarcane. Transgenic sugarcane plants overexpressing *TPS* and *TPP* genes showed increased TRE activity, whereas no changes were observed in transformants containing an RNAi transgene specific for *TRE*^79^. Nevertheless, there is essentially no information about how T6P signaling operates in sugarcane or its potential impact on sucrose accumulation.

All genomes analyzed so far contain genes coding for those enzymes, indicating that trehalose metabolism has ancient origins^14^. When filtered sequences were used for rebuilding the phylogenetic trees of TPS and TPP^15,16^, sugarcane TPS proteins were classified into Class I and Class II clades and their respective subclades (Figs. 3 and 4). Most diploid plants have only one TPS Class I gene, except for paleopolyploid species such as *Z. mays* and poplar (*Populus trichocarpa*), which have two^14^. Four sugarcane sequences from three datasets were grouped in TPS Class I, suggesting a similar pattern observed for the other paleopolyploid species (Fig. 3). These sequences are present in the subclades B1 and B2 that contain all the catalytically active proteins from *A. thaliana* (AtTPS1, AtTPS2, and AtTPS4), *O. sativa* (OsTPS1), and maize (ZmTPSI.1.1). An evaluation of the catalytic residues showed that all amino acids involved in catalysis and binding are present at the sugarcane sequences allocated at the B1 subclade (Class I), indicating that these enzymes are likely to be active and physiologically relevant (Table 3 and Supplementary Fig. S3).

Differently from AtTPS1, the predicted AtTPS2-4 proteins lack the N-terminal auto-inhibitory domain and appear to be restricted to the Brassicaceae^14^, in which these sequences constitute the subclade B2 (Table 2 and Fig. 3). Most of the filtered sugarcane TPS sequences (~ 94%) had high similarity with Class II proteins, reflecting more involvement in regulatory rather than catalytic function as found for most plant species studied to date. Similar results have been recently reported by ^80^, who used phylogenetic trees to classify one sugarcane sequence as possibly catalytic and eight as regulatory.

Unlike TPS, all TPP encode active enzymes^28^, classified in clades A and B and their respective subclades (Fig. 4)^15,16^. In the subclades A1 and B1, various eudicot and monocot species were present, and A2, A3, and B2 were exclusive for monocots^16,58^. Sugarcane sequences were grouped in all subclades, but B1 displayed the highest number of sequences (~ 37%) (Fig. 4). Differently from TPS and TPP, TRE is encoded by a single gene in Arabidopsis and rice, and among 29 sequences retrieved from sugarcane databases, three displayed high similarity with the protein domain template (Supplementary Table S1 and Supplementary Fig. S2).

Multigenic families encode plant TPS and TPP through duplication events which started earlier for TPS than for TPP genes ^15^. Regarding the similarity percentage among sequences belonging to a specific multigenic family, the highest identities for TPS and TPP sequences in maize are 97 and 90%, respectively (data not shown). For filtered sugarcane sequences, 26 TPS, 60 TPP, and three TRE showed less than 97% of identity (Fig. 5), reflecting the existence of distinct predicted homo(eo)logous sequences, including alternative allelic versions of gene products, alternatively spliced variants, and possible paralogs. T6P is an essential regulator of sucrose in plants^84^, and changes in their quantity can modify gene expression and metabolism, maintaining sucrose levels within an optimal range^81^. The in silico expression analysis indicates a variable TPS and TPP gene expression profile in leaves of the sugarcane cultivar SP80-3280 (Fig. 6a and b).

For TPP, subcellular localization was used to identify all *AtTPP* cell- and tissue-specific expression patterns, suggesting neofunctionalization after gene duplications^28^. Our results showed that the sugarcane TPP sequence with the highest expression value belongs to the clade B1 (Fig. 6b). *AtTPPD* and *AtTPPI*, belonging to this subclade, have been associated with abiotic features such as salt and oxidative stress resistance and responses to drought, respectively^82,83^.

For TPS, the highest expression value belongs to a TPS Class I (Fig. 6a), which might indicate that the TPS catalytic function in mature leaves is more relevant. It remains to be elucidated whether the high TPS expression would contribute to high T6P levels. Moreover, sugarcane TPS Class II sequences belonging to subclades A2 and A3 also had high expression values (Fig. 6a). Subclades A2 and A3 also contain AtTPS6 and AtTPS7, which regulate plant architecture, cell shape, and trichome branching^84^. Besides, they are thought to be related to signal transduction during stress resistance^62^. The functional characterization of these sequences in sugarcane might help to elucidate their molecular mechanisms.

The TPS Class I and II duplications led to the neofunctionalization of sequences in a determined clade^10^, which is reflected by mutations at important residues. For TPS Class I, the residues involved in binding and catalysis were maintained in the TPS domain compared with well-characterized sequences. Otherwise, sugarcane TPS Class II sequences showed mutations at residues involved in catalysis, which could explain the acquired regulatory function (Fig. 8, Table 3, and Supplementary Figure S3). The catalysis residues R9 and G22 were conserved in Class I and mutated in Class II sequences (Fig. 3). These residues are important for binding with glucose-6-phosphate and UDP, respectively^66^.

Likewise, the TPP domain of TPS sequences was also analyzed (Table 3). *C. albicans* Tps2 maintains the catalytic activity associated with the TPP domain^66^. Comparing sugarcane and *C. albicans* TPP domains led to identifying many residue mutations associated with catalysis and binding functions (Table 3). Residue replacements at D25G and R67S indicate the complete loss of enzyme activity, which can explain why the TPP domain at sugarcane TPS Class I is probably inactive^66^. Moreover, TPS Class II presented replaced residues involved in binding at the TPP domain (Table 3), mainly at R67K. The replacement of this residue by alanine at *C. albicans* TPP inactivated this phosphatase^66^.

Motif analysis in the TPS proteins was performed to identify specific protein regions associated with a regulatory or catalytic function (Fig. 7). The *Z. mays* genome encodes two Class I TPS sequences (Fig. 3). However, only one (ZmTPSI.1.1) is functional and has all conserved TPS motifs^16^. The second isoform (ZmTPSI.1.2) is truncated and does not have some of the residues necessary for substrate binding^16^. Some studies showed different amounts of TPS motifs, 6 in sugarcane^80^, 20 in potato^62^, and 12 in cotton^85^. Motif 9 (motif 2 in^80^) and motif 8 are present in the TPS domain of TPS Class II of sugarcane and cotton^85^. Together, these results associated with the residue mutations may point out differences in the TPS proteins that may justify the absence of catalytic activity in the regulatory sequences and the centralization of the catalysis in some members of this multigenic family (Table 3). However, further studies are required to validate this hypothesis.

Post-translational modifications have already been experimentally shown to influence TPS Class I sequences’ activity and catalytic fidelity^86^. Phosphorylation at Ser827 and Ser941 and putative SUMOylation at Lys902 were identified in the TPP domain. The latter occurs inside a consensus sequence highly conserved in Class I TPS enzymes in all the major land plant groups and streptophyte algae^86^. *N*-glycosylation is one of the most common and chemically complex post-translational modifications in eukaryotes^87^. However, there is little information for TPS and TPP families members. Sugarcane TPS Class I and II showed 17 and 41 putative *N*-glycosylation sites, respectively (Supplementary Table S3). Although these sites do not provoke significant changes in protein structure, they might influence the dynamic properties, protein stability, and possibly the enzyme’s catalytic activity^87^.

Genetic manipulation of the trehalose pathway improves tolerance to different abiotic stresses^20,88^. Sugarcane is an annual crop cultivated in large geographical areas worldwide, facing constant environmental changes such as temperature and water availability. Water deficit differentially affects sugarcane during the distinct growth stages and is considered one of the main factors limiting its productivity^89–93^. However, water deficit is beneficial to enhance the influx of sucrose into the stems during the maturation phase^94^. Thus, a better understanding of the processes mediated by the trehalose pathway in sugarcane is also an alternative to mitigate current environmental pressures derived from climate change and boost sugarcane-derived products’ production. As a large sugar and bioethanol producer, any modest gain in sugarcane productivity in Brazil represents significant profits for the bioenergy sector.

## Conclusions

The role of T6P as a sucrose sensor is well known. However, the involvement of trehalose metabolism reported so far for sugarcane recognized it as a putative mediator of osmoprotectant mechanism under stress. The high sucrose levels in sugarcane stems could indicate a role for T6P as a central regulator during sugarcane growth and development. This study uncovered a large number of sequences with high homology to the selected target genes. However, the exact numbers of TPS, TPP, and TRE sequences in sugarcane are not yet precise as even the most complete database was unable to cover the entire sugarcane genome. Apart from classifying TPS and TPP proteins from sugarcane into distinct clades, amino acid residue and motif analyses revealed specific alterations contributing to a catalytic or regulatory function. We managed to retrieve expression values from one sugarcane transcriptome dataset, but more information is needed to map under what conditions and in which tissues these genes are expressed. Our findings started to pave the way for functional studies to uncover the physiological roles of T6P signaling in sugarcane.

## Material and methods

### Identification of trehalose metabolizing enzymes in sugarcane

Gene sequences encoding trehalose metabolizing enzymes from *A. thaliana*, *Z. mays*, and *O. sativa* were first used as queries to identify the groups of orthologous genes they belonged to at the level of Viridiplantae, in the EggNOG v4.5.1 database^54^. The orthologous group (OG) IDs were then used to identify genes belonging to the same groups in species of the subfamily Panicoideae, whose genomes are publicly available. For sugarcane, a mix of genomics and transcriptomics datasets was used^47,49,52,53,55,56^ (Table 1).

### Sequence alignment and phylogenetic analyses

To determine the percentage of identity among all filtered TPS and TPP protein sequences retrieved from different sugarcane databases, a global pairwise alignment among all sequence pairs was carried out with the program needle of the EMBOSS v6.0.0. suite^95^. Additionally, multiple sequence alignments for the protein sequences of each OG were generated with MAFFT ^96^, and dubious regions were removed from the alignments using TrimAI v1.2^97^.

Phylogenetic inference was performed by IQ-TREE v1.6.9^59^, with automatic evolutionary model selection and branch support values were computed as Shimodaira–Hasegawa approximate likelihood ratio test (SH-like aLRT)^60^, and Ultrafast bootstrap (UFboot)^61^. Phylogenetic trees were rooted by reconciling them with the commonly accepted species tree with Notung v2.9^98^. Lastly, the phylogenetic trees of filtered TPS and TPP sugarcane sequences were constructed based on^15^ and^16^, respectively, using the above settings.

### Domain characterization, three-dimensional protein structure analyses, and catalytic/binding residues

Protein sequences of *A. thaliana*, *S. bicolor*, and sugarcane were subjected to domain analysis using HMMER v2.41.2^57,99^. Sequences that matched with a score above the gathering threshold and covered at least 80% of the domain model were considered for further analyses. This threshold reflected the domain coverage value, representing how much of the domains were detected in sugarcane sequences. Sequences harboring all the predicted conserved domains were illustrated using the Illustrator for biological sequences v1.0^100^. The three-dimensional structures were modeled by SWISS-MODEL^63^. The best template of each sequence was selected, combining larger sequence coverage, global model quality estimation (GMQE), quaternary structure quality estimate (QSQE), and the sequence identity to the target. The obtained structures were processed using PyMol (TM) 2.4.2, and the catalytic and binding residues were identified at modeled structures by the alignment with TPS from *E. coli*^64,66^, as well as TPS and TPP from *C. albicans*^65,66^.

### In silico transcript expression patterns

TPS, TPP, and TRE expression levels were recovered from a published transcriptomics dataset of leaf development from the hybrid SP80-3280^56^. As most of the databases were from hybrid cultivars, and to avoid errors in the analysis, the sequences of the *S. spontaneum* have been removed from this analysis. In^56^ different developmental regions along the leaf + 1 of two-month-old seedlings were evaluated but we focused on samples from the middle portion (4 biological replicates), as this is the most photosynthetically active region (NCBI Short Read Archive accession numbers: SRR1979669, SRR1979665, SRR1979662, and SRR1979660). Raw sequence reads were downloaded from NCBI’s SRA, and cleaned with BBDuk2^101^, to remove remainders of rRNA and low-quality regions as well as adapters. Salmon v1.1.0^102^ was used to estimate transcript expression levels expressed as Transcripts per Million (TPM) transformed as log_10_(TPM + 1).

### Prediction of *N*-glycosylation sites and conserved motifs

The potential *N*‐glycosylation (Asn) sites from TPS Class I and II filtered sequences were predicted with NetNGlyc 1.0 software^103^. As the software recommended, only *N*‐glycosylation sites prediction with potential values > 0.5. Subsequently, to identify the motif regions, the two sugarcane sequences with the highest expression values of each clade of the TPS Class I and II were submitted to MEME^104^, using default parameters and the maximum number of motifs set to 12. For this analysis, were used sequences of *A. thaliana*, rice, maize, and a basal green alga, *O. tauri*. A worflow that summarizes all the steps followed in this article is in Supplementary Fig. S4.

## Supplementary Information

Supplementary Information 1.

Supplementary Information 2.

Supplementary Information 3.
**Figure S3.** Multiple protein sequence alignment of TPS Class I and II, horizontal red boxes indicate the TPS Class I sugarcane sequence with the highest expression value (SCA1_2_Sh239M11_p000040), as well as E. Coli TPS (CAA48913.1) and C. albicans Tps1 (5huu). Horizontal black boxes indicate the TPS Class II sugarcane sequence with the highest expression value (SCA3_SP803280_c117830_g1_i1_m154722) and C. albicans Tps2 (XP_721536.1). Vertical red and black boxes indicate residues involved in binding and catalysis of TPS Class I and II, respectively. All residues and possible mutations are described in Table 3.

Supplementary Information 4.

Supplementary Information 5.

Supplementary Information 6.

Supplementary Information 7.

## Data Availability

The datasets supporting the conclusions of this article are included within the article and its additional files.
