# Supplementary material for: Bioinformatic analyses to uncover genes involved in trehalose metabolism in the polyploid sugarcane
Source: Sci Rep. 2022 May 7;12:7516. doi: 10.1038/s41598-022-11508-x (PMC9079074; doi:10.1038/s41598-022-11508-x)
Supplement: Supplementary file 1 — Supplementary Information 1. [file 41598_2022_11508_MOESM1_ESM.docx]

1.
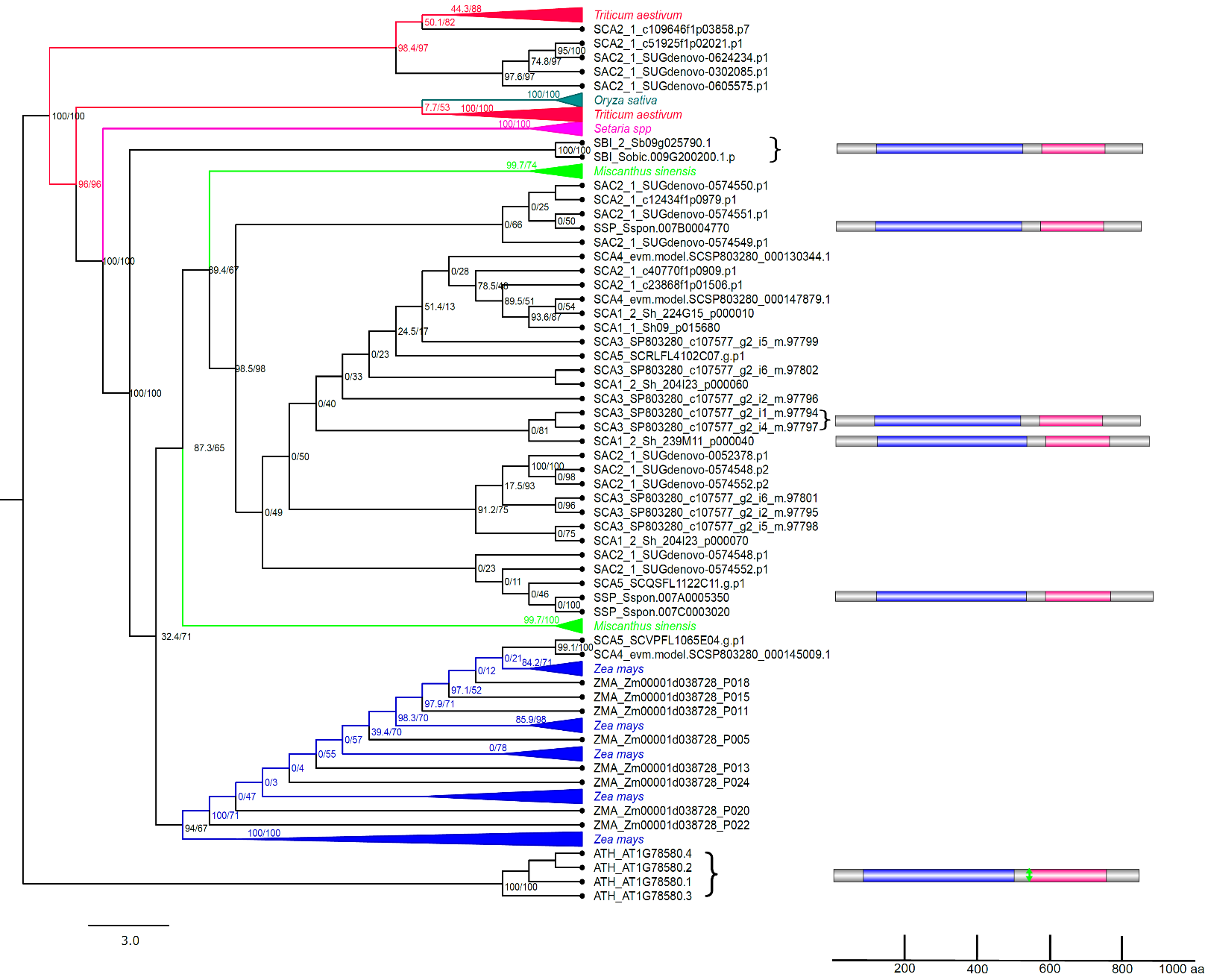
**1DVBM (OG) - TPS
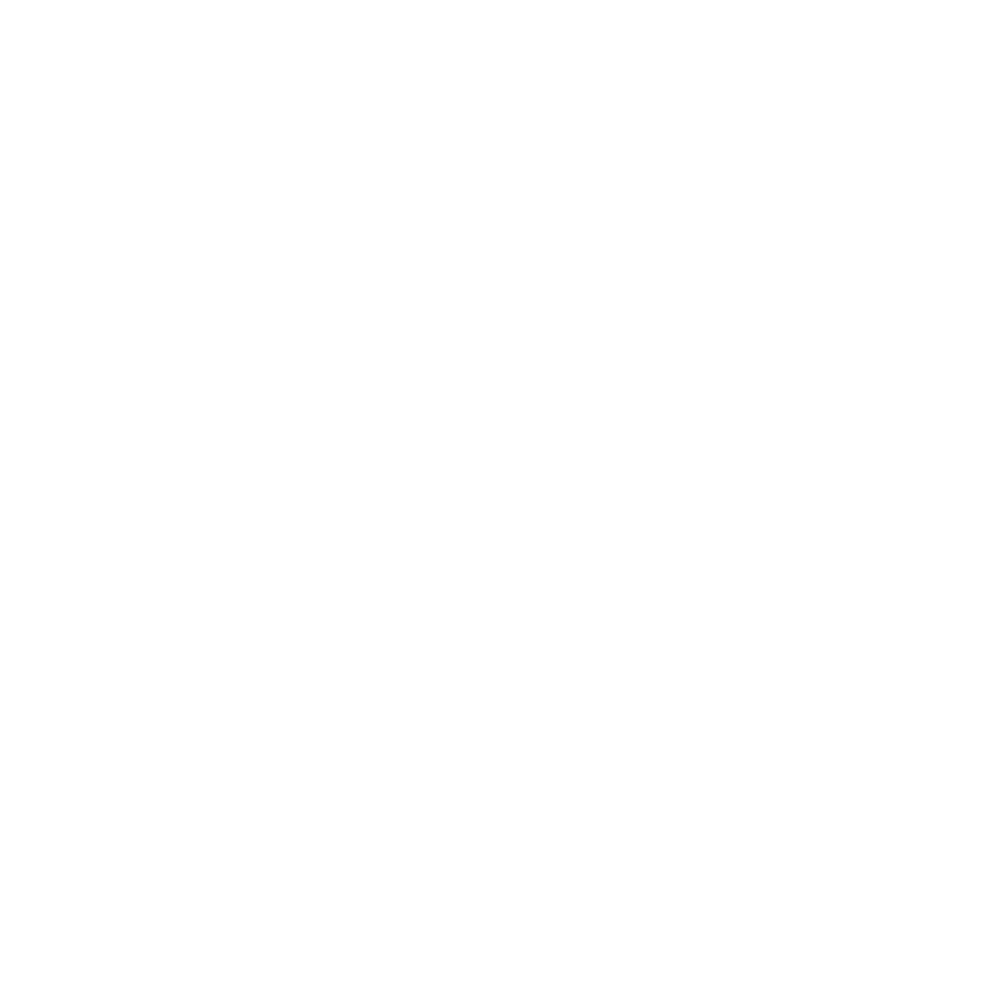
**

**Figure S1. a) Phylogenetic tree for the OG 1DVBM containing trehalose-6-phosphate synthase (TPS) sequences.** Phylogenetic trees were constructed from publicly protein sequences of *A. thaliana*, *Miscanthus sinensis, O. sativa, Setaria spp*, *S. bicolor,* and *Z. mays* datasets. The trees were built with IQ-TREE [59] using automatic evolutionary model selection and ultrafast bootstrap approximation (UFboot) [61] with branch support values improved by the approximate likelihood ratio test (SH-like aLRT %) [60]. Their respective domains were annotated in HMMERscan, domains glycosyltransferase and trehalose-phosphatase are represented in blue and pink, respectively. Predicted active sites are shown in green. The scale represents the number of amino acids in each protein. Databases and accession numbers are listed in Supplementary Table S1.

1. **1DQAR (OG) – TPS**


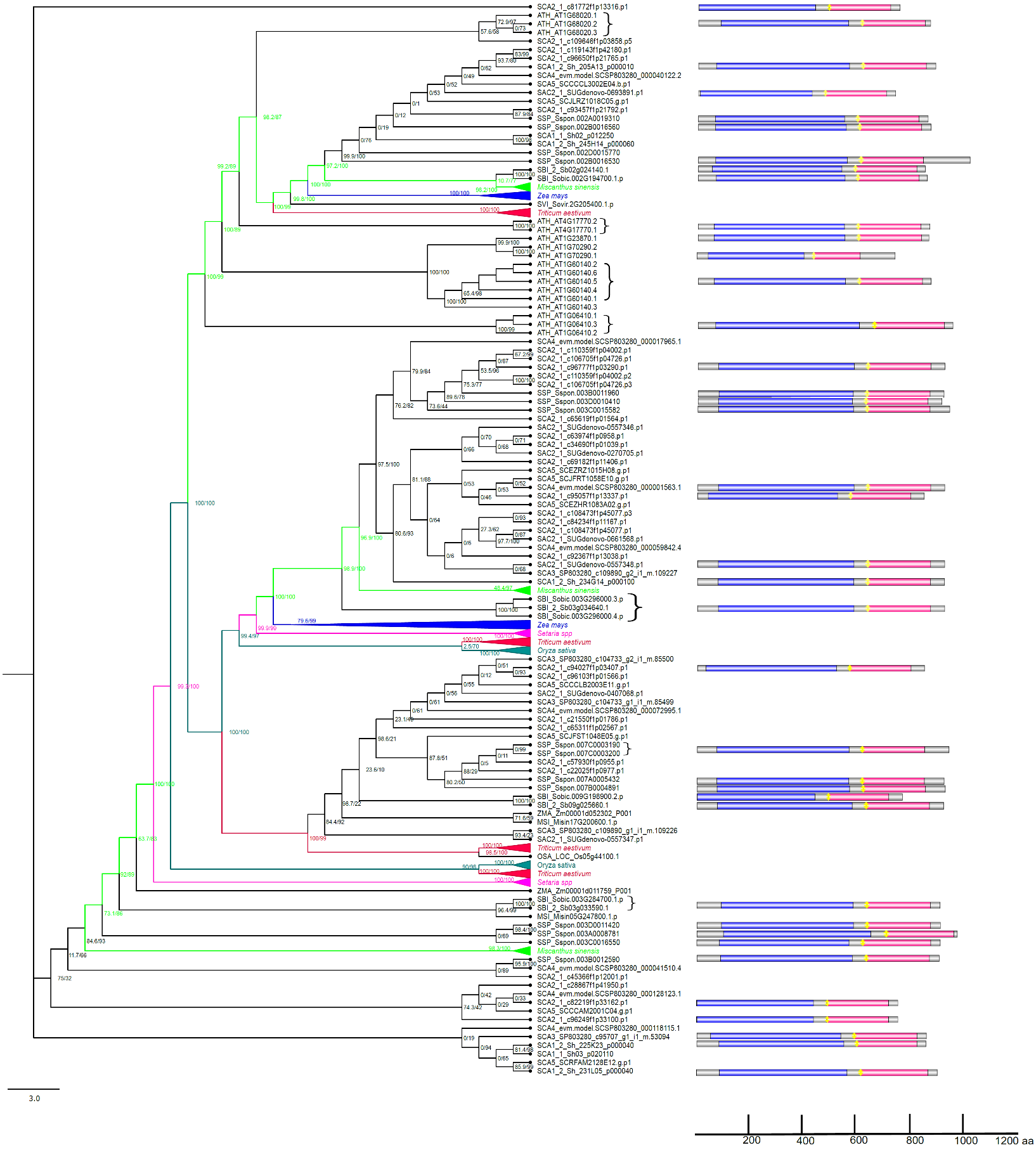


-
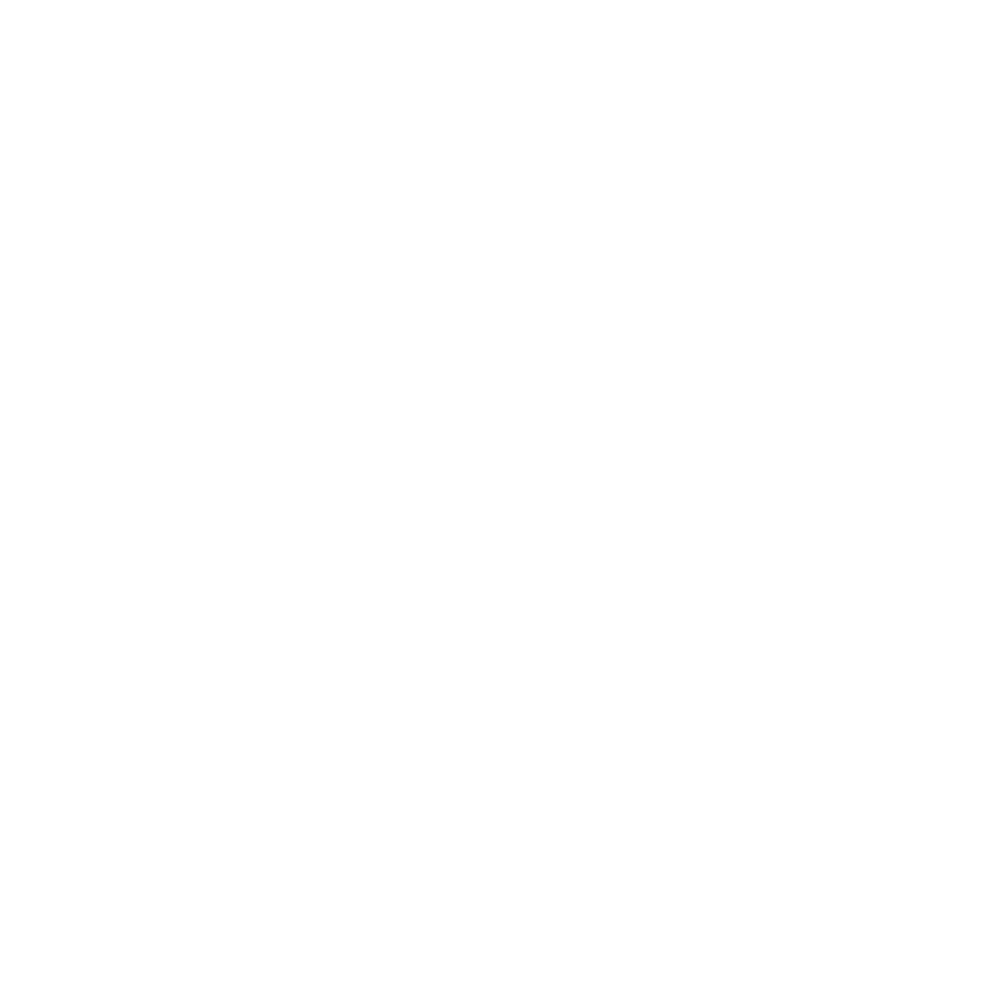

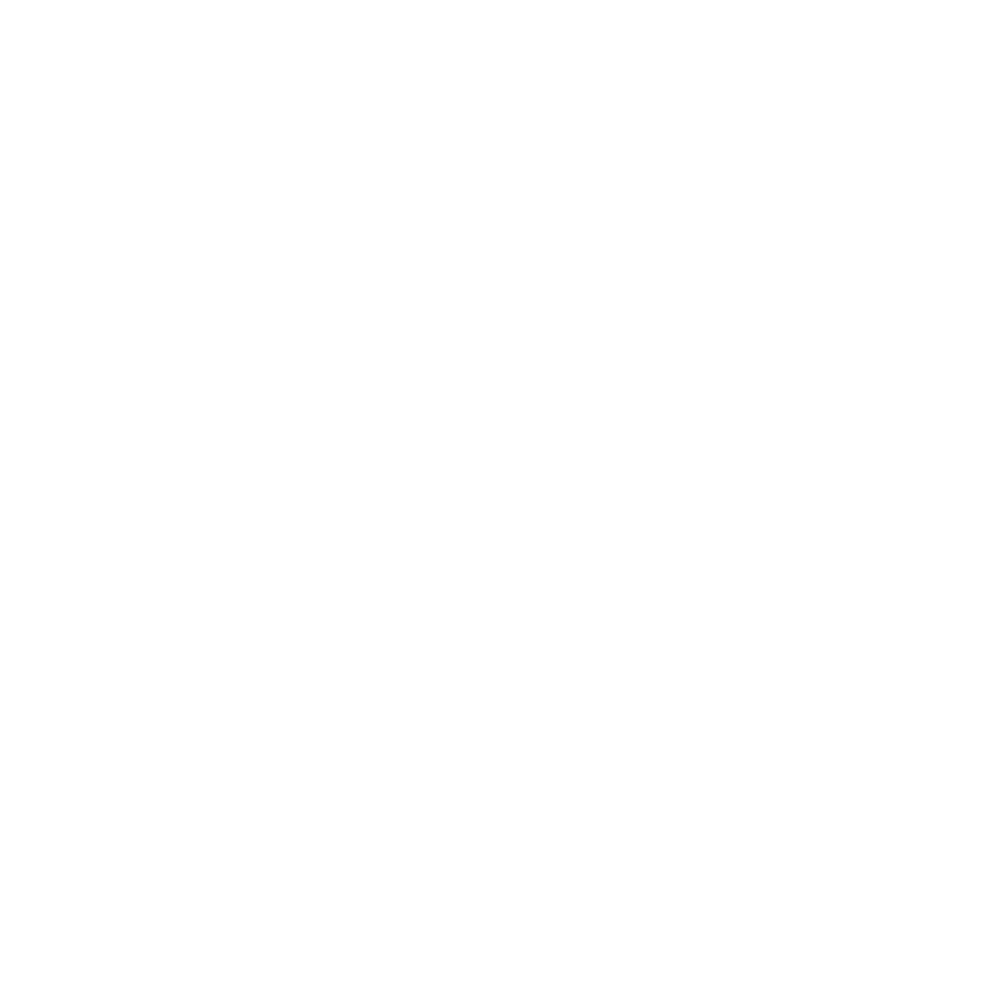

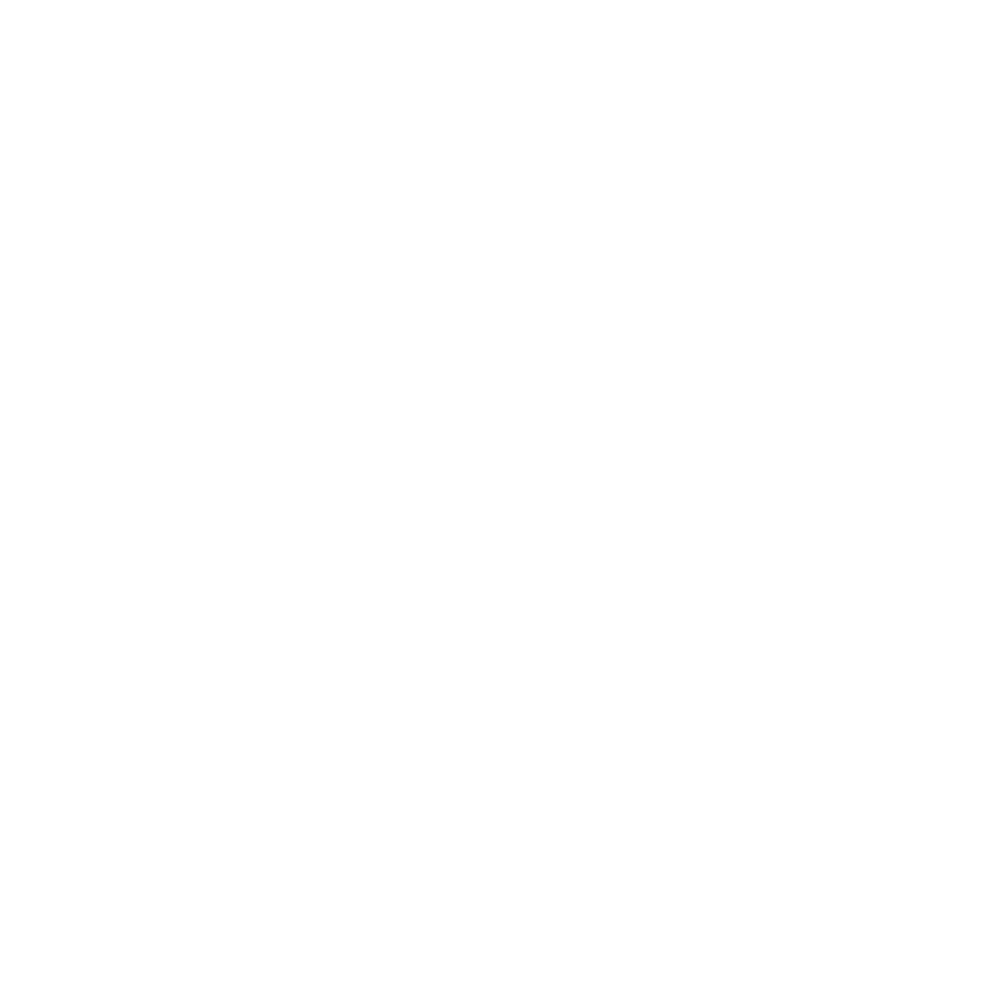

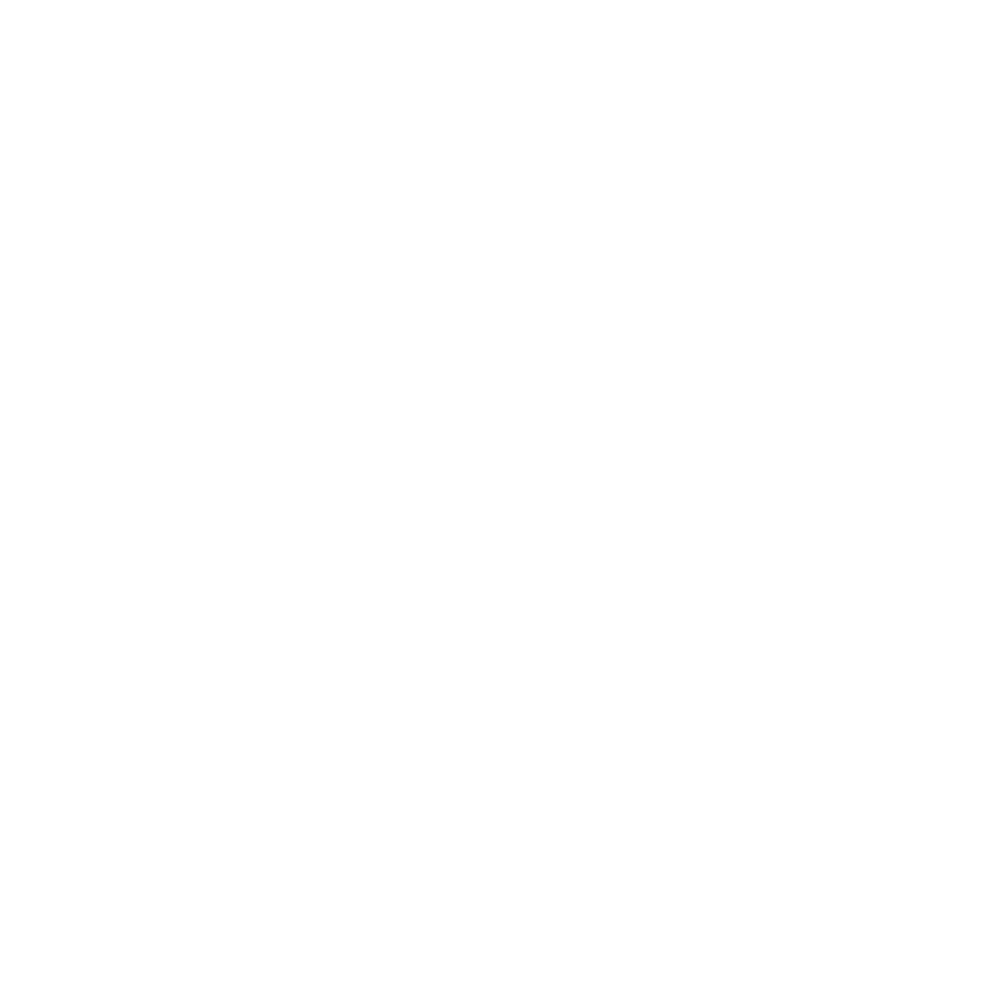

- **Figure S1. b) Phylogenetic tree for the OG 1DQAR containing trehalose-6-phosphate synthase (TPS) sequences.** Phylogenetic trees were constructed from publicly protein sequences of *A. thaliana*, *Miscanthus sinensis*, *O. sativa*, *Setaria* spp, *S. bicolor*, and Z. mays datasets. The trees were built with IQ-TREE [59] using automatic evolutionary model selection and ultrafast bootstrap approximation (UFboot) [61] with branch support values improved by the approximate likelihood ratio test (SH-like aLRT %) [60]. Their respective domains were annotated in HMMERscan, domains glycosyltransferase and trehalose-phosphatase are represented in blue and pink, respectively. Predicted active sites are shown in yellow. The scale represents the number of amino acids in each protein. Databases and accession numbers are listed in Supplementary Table S1.

1.
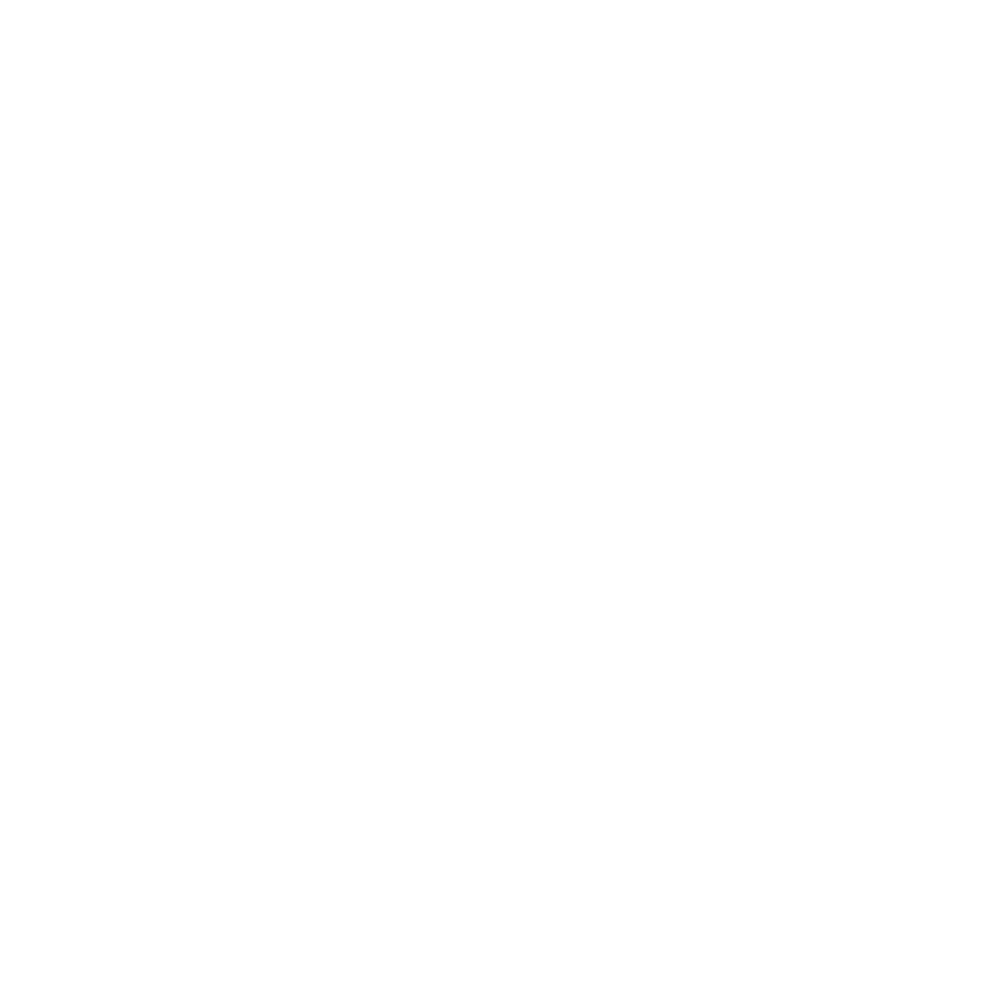

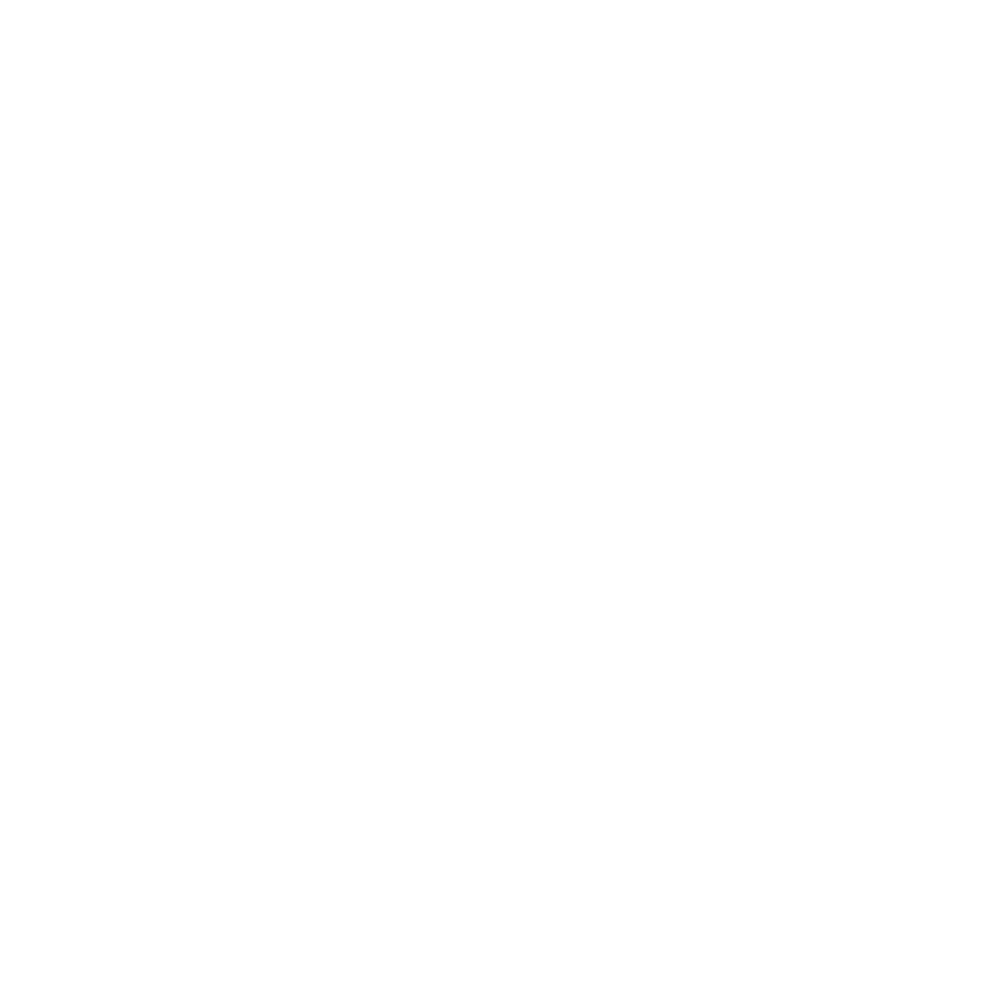

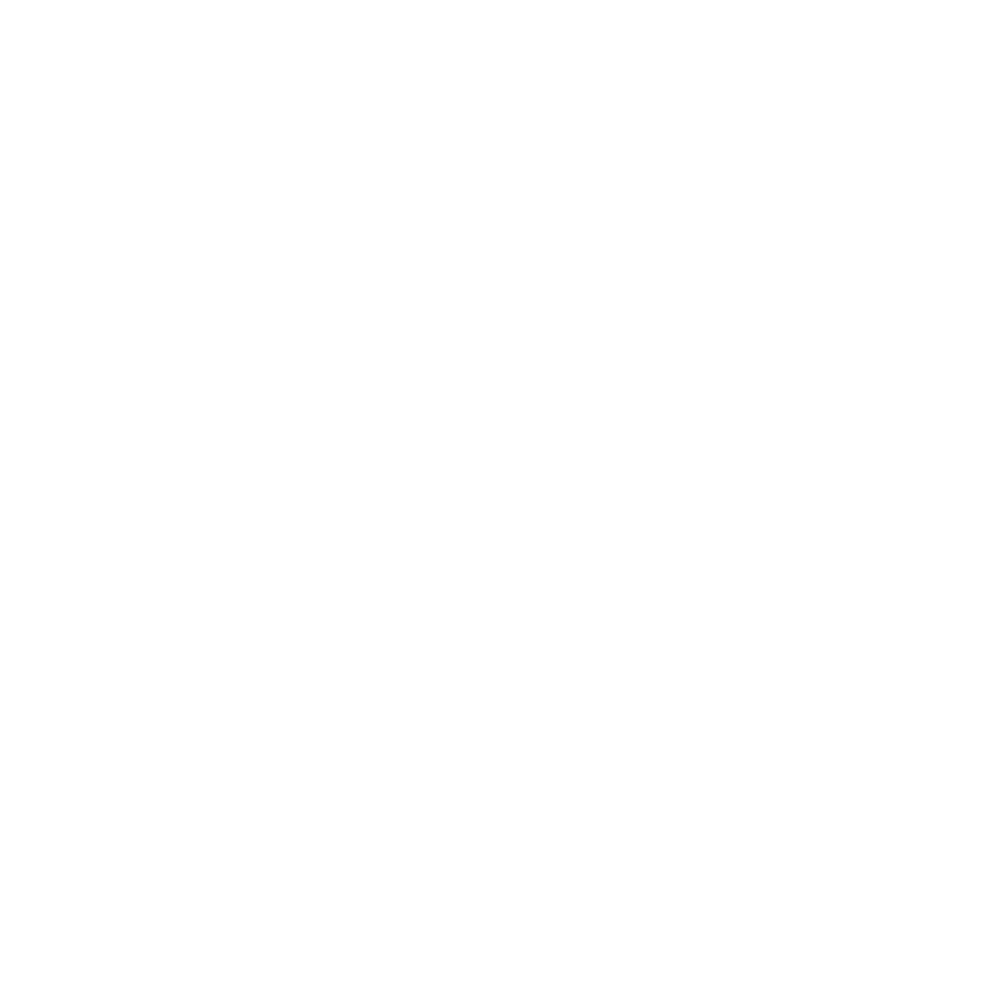

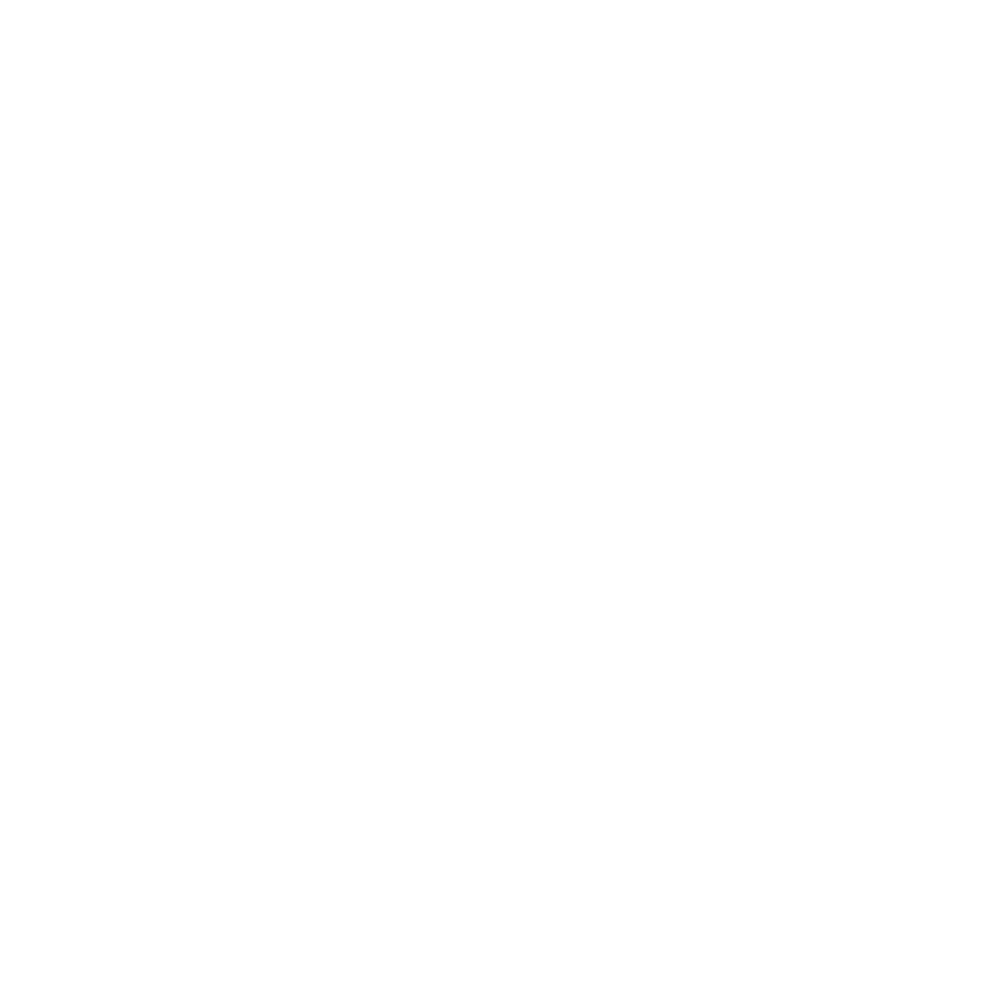

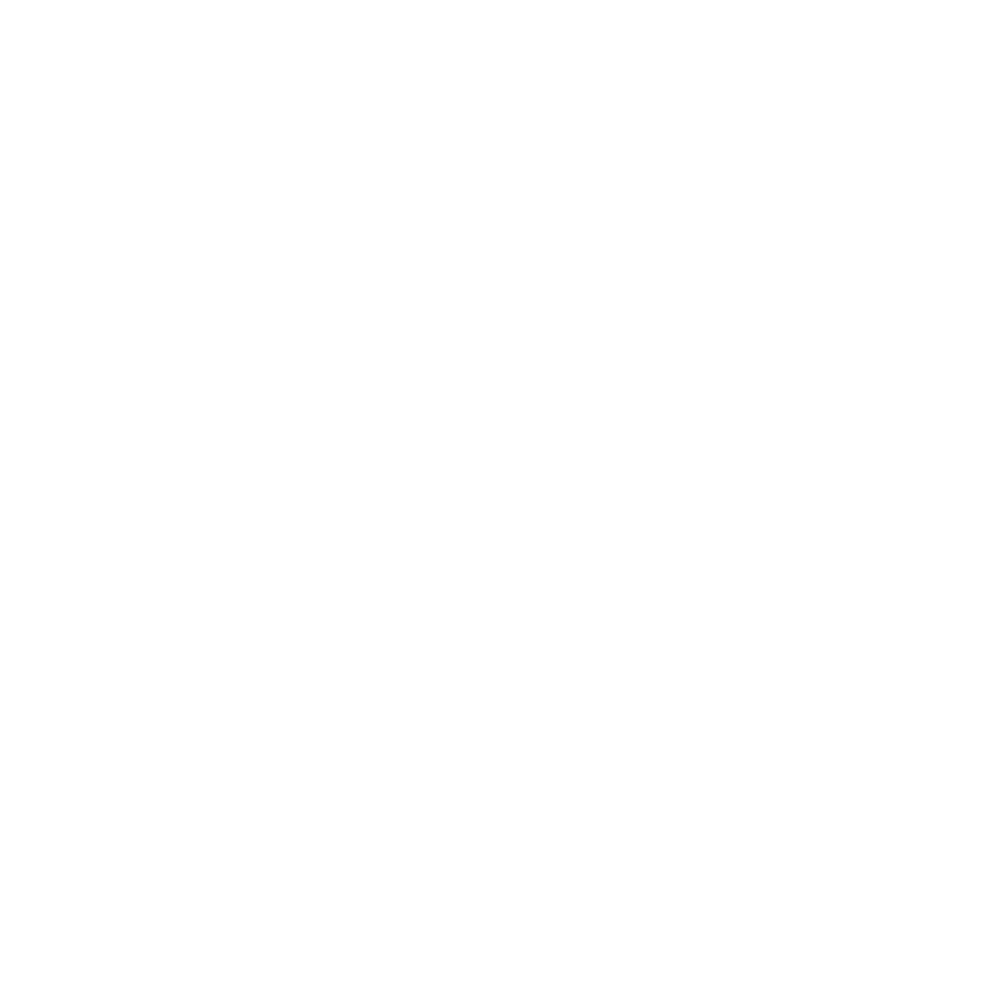

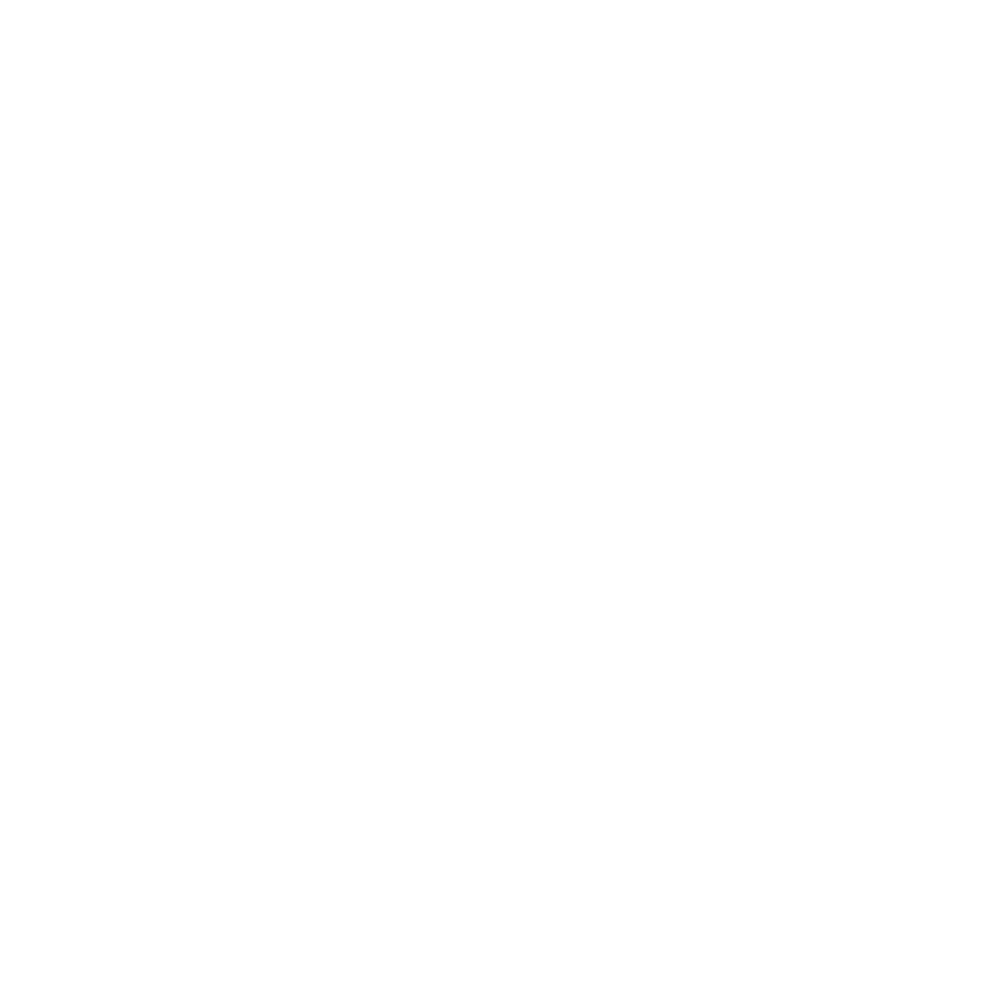

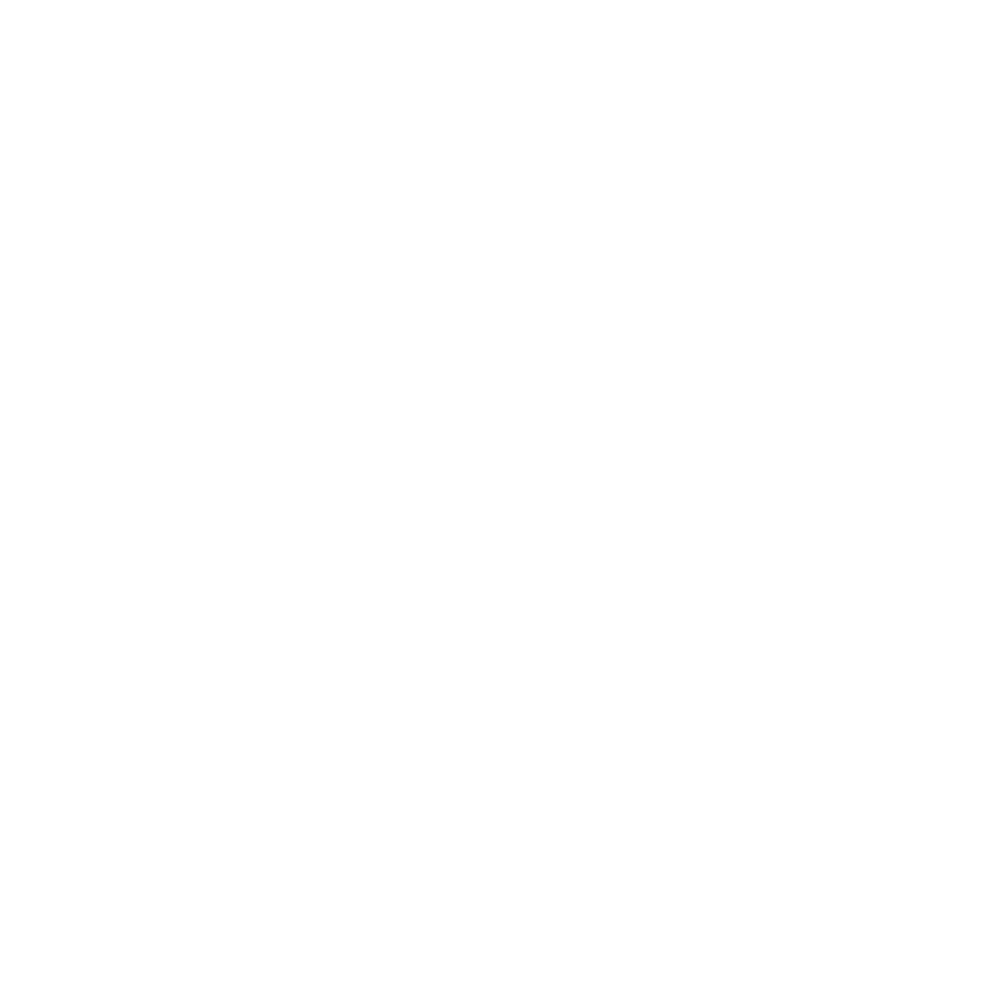
**1DSVD (OG) – TPS**


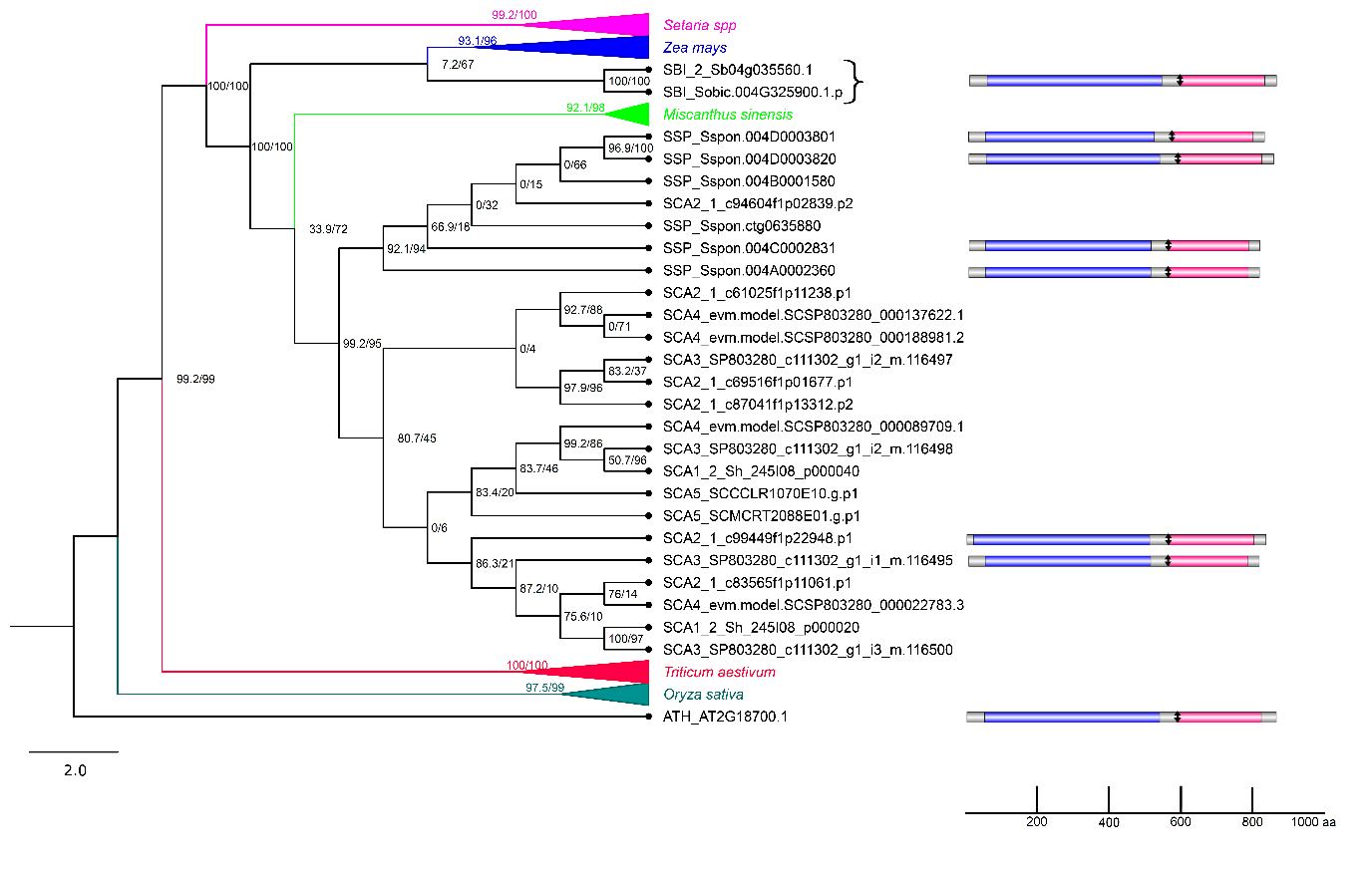


**Figure S1. c) Phylogenetic tree for the OG 1DSVD containing trehalose-6-phosphate synthase (TPS) sequences.** Phylogenetic trees were constructed from publicly protein sequences of *A. thaliana*, *Miscanthus sinensis*, *O. sativa*, *Setaria* spp, *S. bicolor*, and *Z. mays* datasets. The trees were built with IQ-TREE [59] using automatic evolutionary model selection and ultrafast bootstrap approximation (UFboot) [61] with branch support values improved by the approximate likelihood ratio test (SH-like aLRT %) [60]. Their respective domains were annotated in HMMERscan, domains glycosyltransferase and trehalose-phosphatase are represented in blue and pink, respectively. Predicted active sites are shown in black. The scale represents the number of amino acids in each protein. Databases and accession numbers are listed in Supplementary Table S1.

1.
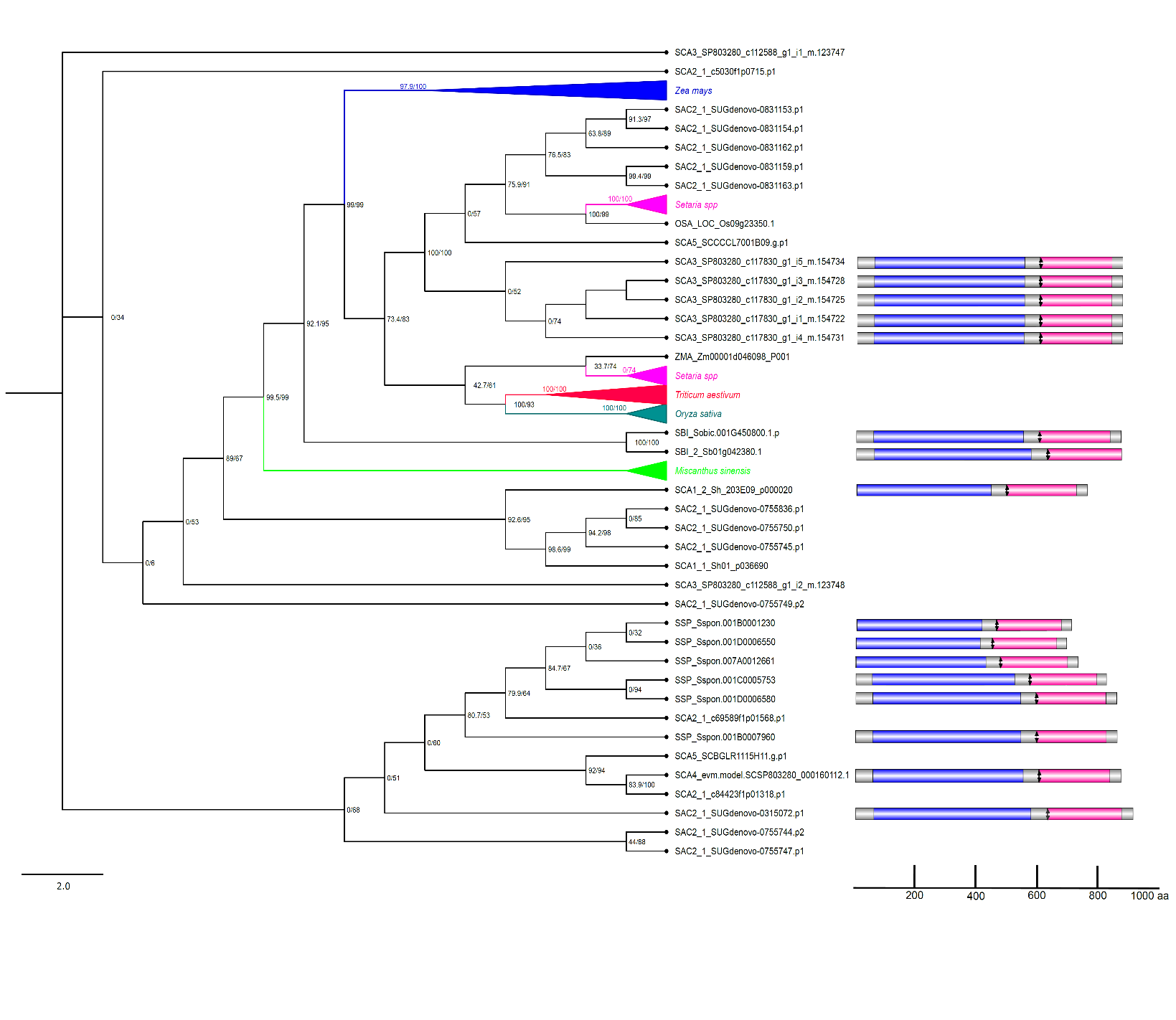
**1DZIJ (OG) – TPS**

**Figure S1. d) Phylogenetic tree for the OG 1DZIJ containing trehalose-6-phosphate synthase (TPS) sequences.** Phylogenetic trees were constructed from publicly protein sequences of *A. thaliana*, *Miscanthus sinensis*, *O. sativa*, *Setaria* spp, *S. bicolor*, and *Z. mays* datasets. The trees were built with IQ-TREE [59] using automatic evolutionary model selection and ultrafast bootstrap approximation (UFboot) [61] with branch support values improved by the approximate likelihood ratio test (SH-like aLRT %) [60]. Their respective domains were annotated in HMMERscan, domains glycosyltransferase and trehalose-phosphatase are represented in blue and pink, respectively. Predicted active sites are shown in black. The scale represents the number of amino acids in each protein. Databases and accession numbers are listed in Supplementary Table S1.

1.
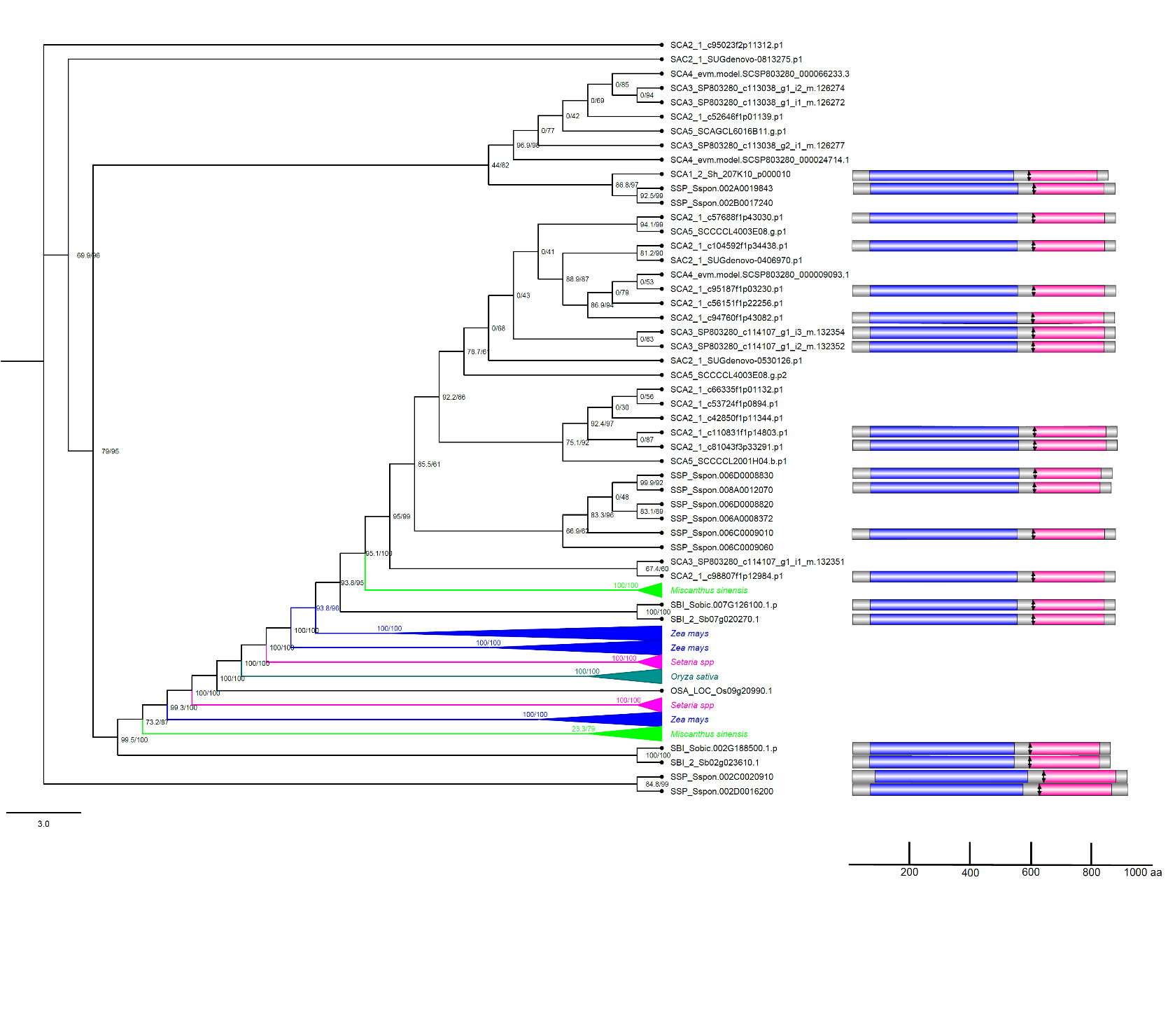
**1E175 (OG) – TPS**

**Figure S1. e) Phylogenetic tree for the OG 1E175 containing trehalose-6-phosphate synthase (TPS) sequences.** Phylogenetic trees were constructed from publicly protein sequences of *A. thaliana*, *Miscanthus sinensis*, *O. sativa*, *Setaria* spp, *S. bicolor*, and *Z. mays* datasets. The trees were built with IQ-TREE [59] using automatic evolutionary model selection and ultrafast bootstrap approximation (UFboot) [61] with branch support values improved by the approximate likelihood ratio test (SH-like aLRT %) [60]. Their respective domains were annotated in HMMERscan, domains glycosyltransferase and trehalose-phosphatase are represented in blue and pink, respectively. Predicted active sites are shown in black. The scale represents the number of amino acids in each protein. Databases and accession numbers are listed in Supplementary Table S1**.**

1.
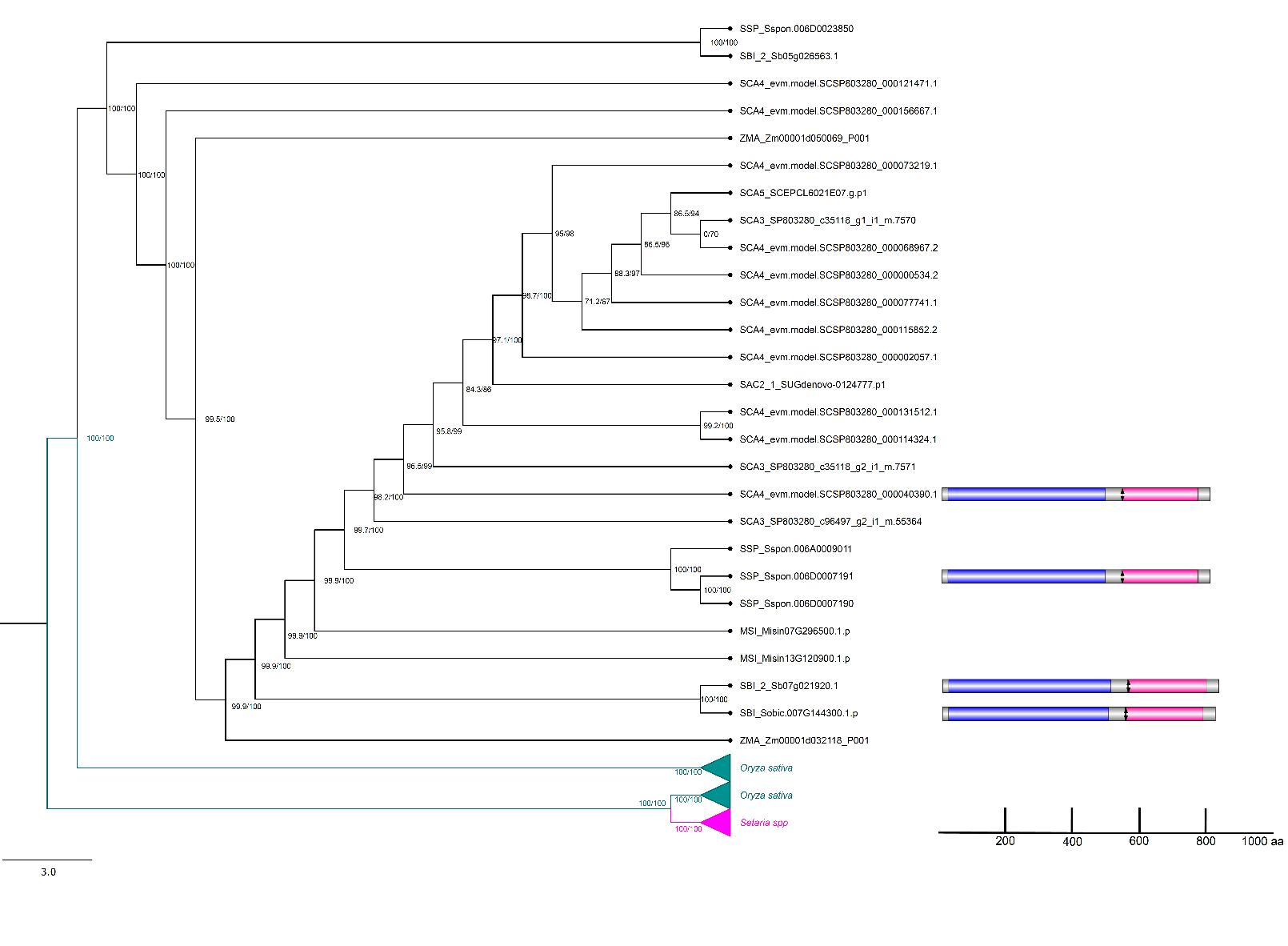
**1DQDD (OG) - TPS**

**Figure S1. f) Phylogenetic tree for the OG 1DQDD containing trehalose-6-phosphate synthase (TPS) sequences.** Phylogenetic trees were constructed from publicly protein sequences of *A. thaliana*, *Miscanthus sinensis*, *O. sativa*, *Setaria* spp, *S. bicolor*, and *Z. mays* datasets. The trees were built with IQ-TREE [59] using automatic evolutionary model selection and ultrafast bootstrap approximation (UFboot) [61] with branch support values improved by the approximate likelihood ratio test (SH-like aLRT %) [60]. Their respective domains were annotated in HMMERscan, domains glycosyltransferase and trehalose-phosphatase are represented in blue and pink, respectively. Predicted active sites are shown in black. The scale represents the number of amino acids in each protein. Databases and accession numbers are listed in Supplementary Table S1.

1.
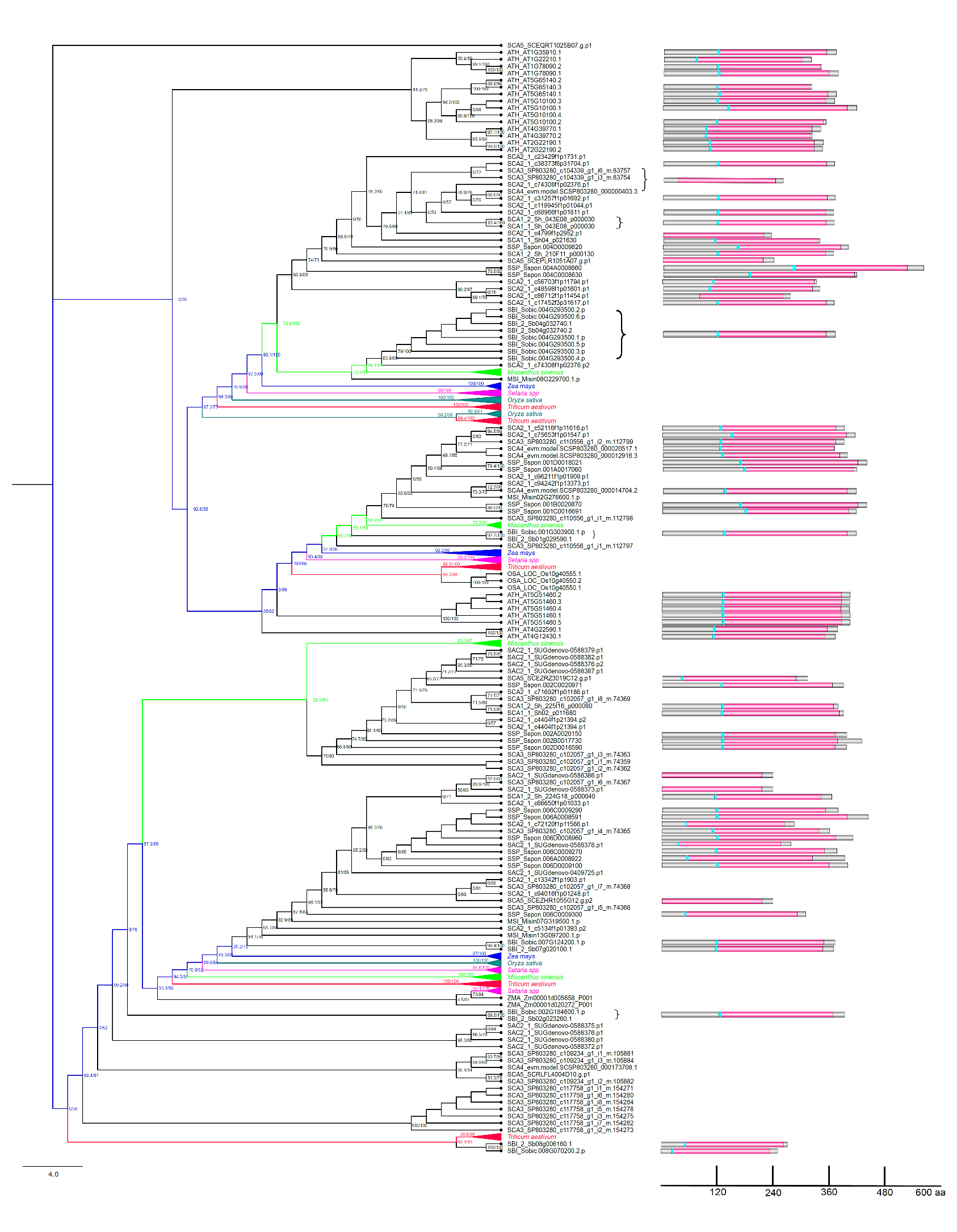
**1EKDT (OG) - TPP**

**Figure S1. g) Phylogenetic tree for the 1EKDT containing trehalose-6-phosphate phosphatase (TPP) sequences.** Phylogenetic trees were constructed from publicly protein sequences of *A. thaliana*, *Miscanthus sinensis*, *O. sativa*, *Setaria* spp, *S. bicolor*, and *Z. mays* datasets. The trees were built with IQ-TREE [59] using automatic evolutionary model selection and ultrafast bootstrap approximation (UFboot) [61] with branch support values improved by the approximate likelihood ratio test (SH-like aLRT %) [60]. Their respective domains were annotated in HMMERscan, domain trehalose-phosphatase is represented in pink. Predicted active sites are shown in blue. The scale represents the number of amino acids in each protein. Databases and accession numbers are listed in Supplementary Table S1.

1.
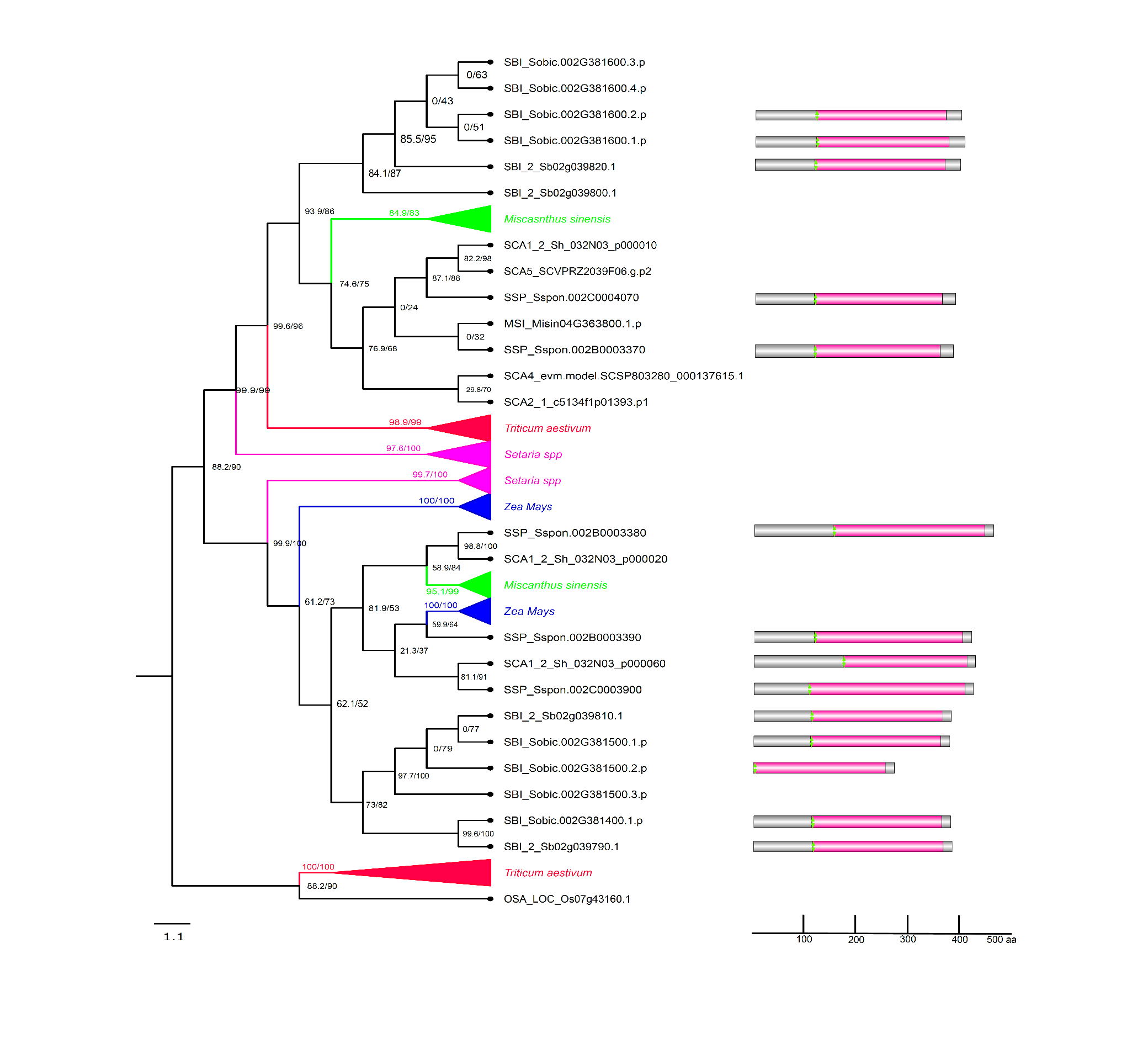
**EKDN (OG) – TPP**

**Figure S1. h) Phylogenetic tree for the EKDN containing trehalose-6-phosphate phosphatase (TPP) sequences.** Phylogenetic trees were constructed from publicly protein sequences of *A. thaliana*, *Miscanthus sinensis*, *O. sativa*, *Setaria* spp, *S. bicolor*, and *Z. mays* datasets. The trees were built with IQ-TREE [59] using automatic evolutionary model selection and ultrafast bootstrap approximation (UFboot) [61] with branch support values improved by the approximate likelihood ratio test (SH-like aLRT %) [60]. Their respective domains were annotated in HMMERscan, domain trehalose-phosphatase is represented in pink. Predicted active sites are shown in green. The scale represents the number of amino acids in each protein. Databases and accession numbers are listed in Supplementary Table S1.

1.
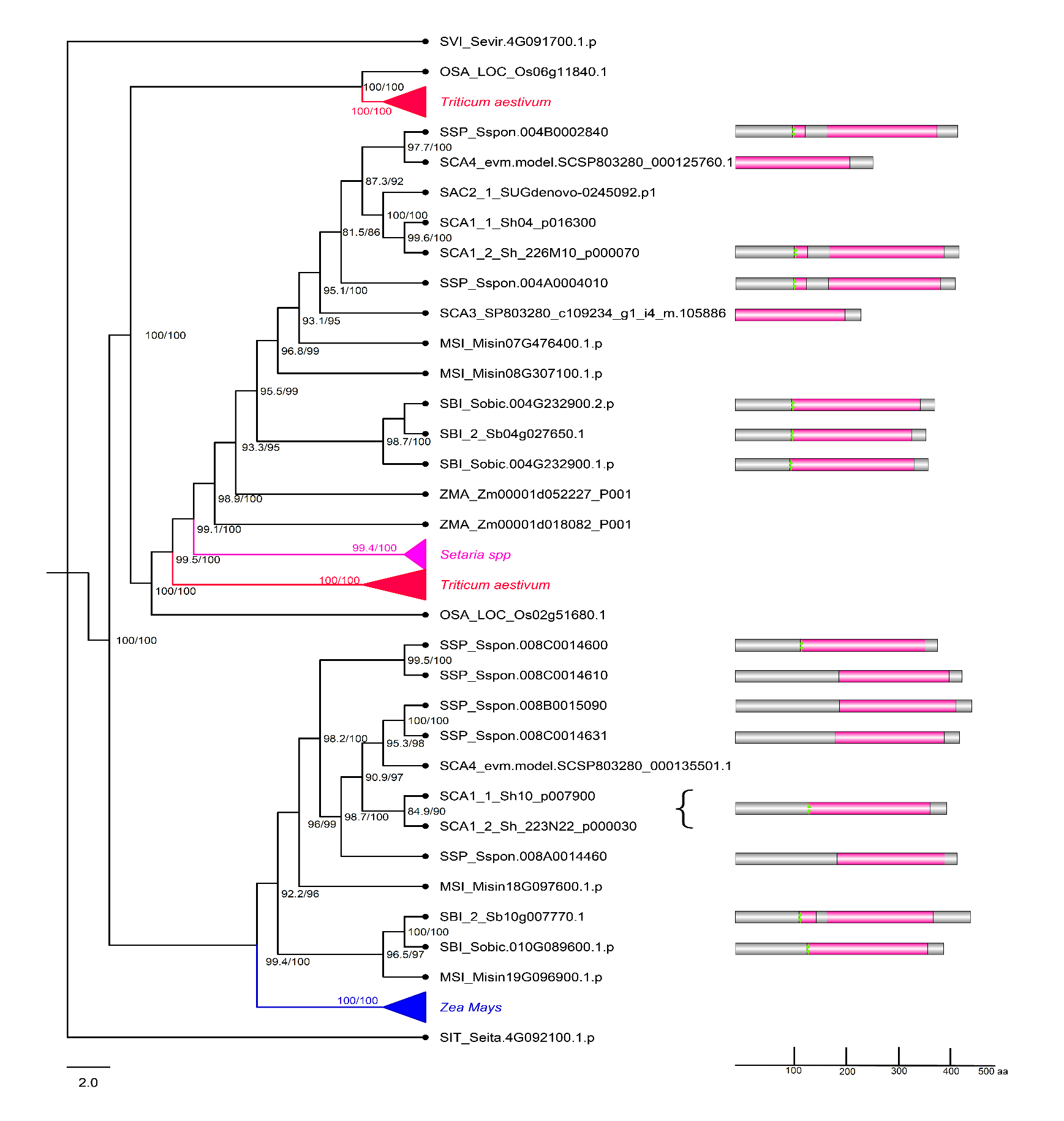
**1EKDR (OG) - TPP**

**Figure S1. i) Phylogenetic tree for the 1EKDR containing trehalose-6-phosphate phosphatase (TPP) sequences.** Phylogenetic trees were constructed from publicly protein sequences of *A. thaliana*, *Miscanthus sinensis*, *O. sativa*, *Setaria* spp, *S. bicolor*, and *Z. mays* datasets. The trees were built with IQ-TREE [59] using automatic evolutionary model selection and ultrafast bootstrap approximation (UFboot) [61] with branch support values improved by the approximate likelihood ratio test (SH-like aLRT %) [60]. Their respective domains were annotated in HMMERscan, domain trehalose-phosphatase is represented in pink. Predicted active sites are shown in green. The scale represents the number of amino acids in each protein. Databases and accession numbers are listed in Supplementary Table S1.

1.
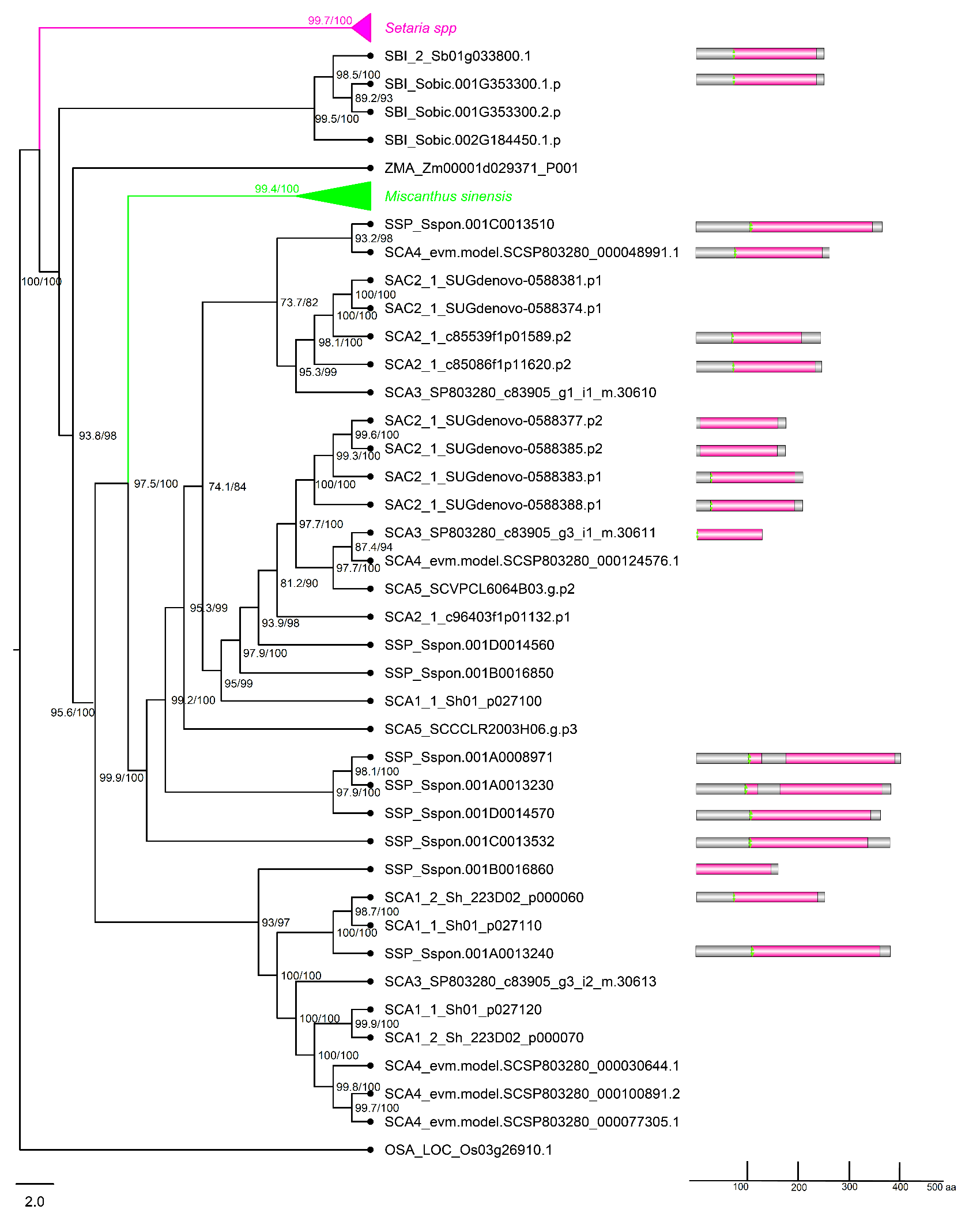
**1EKDP (OG) – TPP**

**Figure S1. j) Phylogenetic tree for the 1EKDP containing trehalose-6-phosphate phosphatase (TPP) sequences.** Phylogenetic trees were constructed from publicly protein sequences of *A. thaliana*, *Miscanthus sinensis*, *O. sativa*, *Setaria* spp, *S. bicolor*, and *Z. mays* datasets. The trees were built with IQ-TREE [59] using automatic evolutionary model selection and ultrafast bootstrap approximation (UFboot) [61] with branch support values improved by the approximate likelihood ratio test (SH-like aLRT %) [60]. Their respective domains were annotated in HMMERscan, domain trehalose-phosphatase is represented in pink. Predicted active sites are shown in green. The scale represents the number of amino acids in each protein. Databases and accession numbers are listed in Supplementary Table S1.

1.
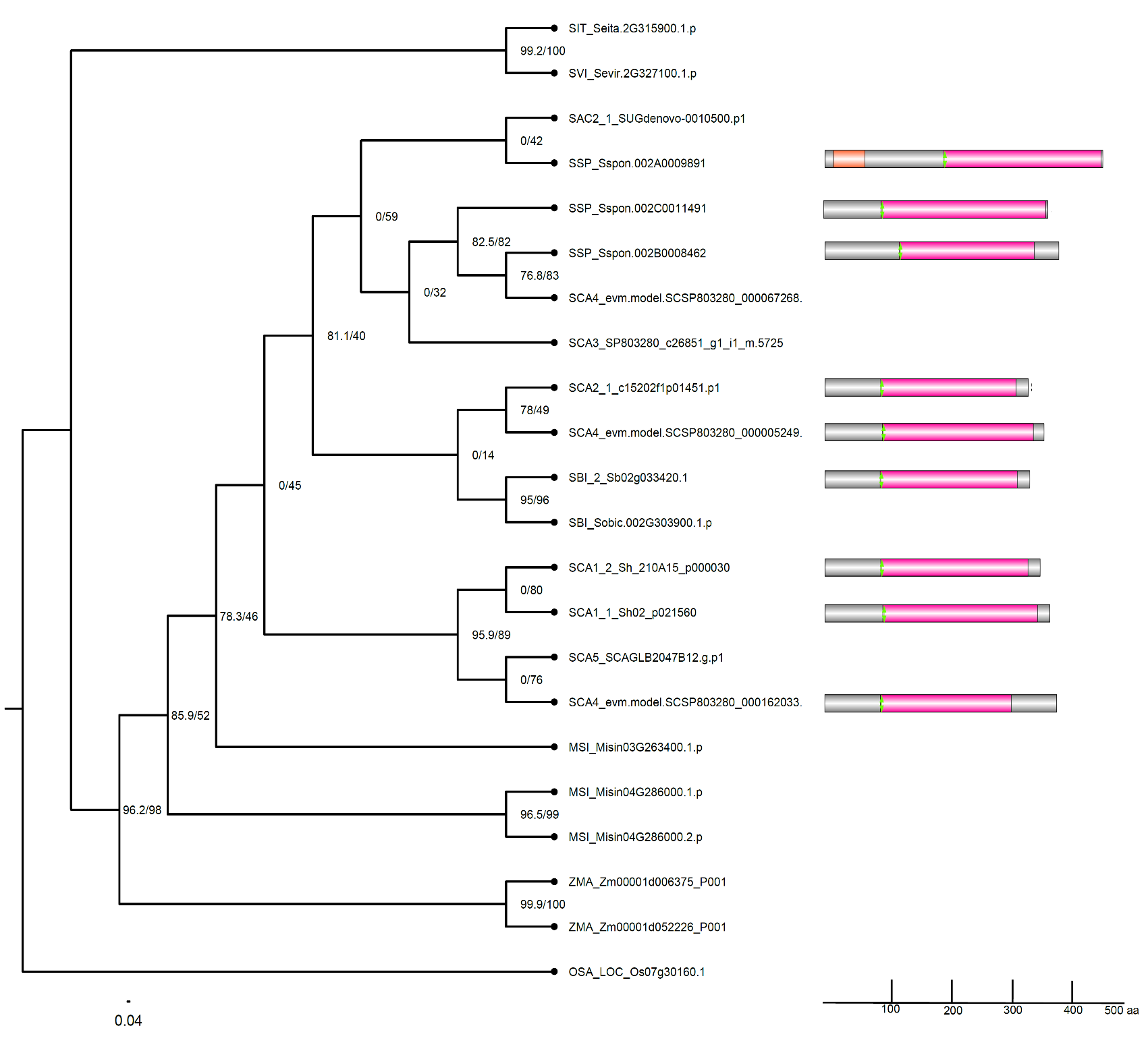
**1EKDS (OG) – TPP**

**Figure S1. k) Phylogenetic tree for the 1EKDS containing trehalose-6-phosphate phosphatase (TPP) sequences.** Phylogenetic trees were constructed from publicly protein sequences of *A. thaliana*, *Miscanthus sinensis*, *O. sativa*, *Setaria* spp, *S. bicolor*, and *Z. mays* datasets. The trees were built with IQ-TREE [59] using automatic evolutionary model selection and ultrafast bootstrap approximation (UFboot) [61] with branch support values improved by the approximate likelihood ratio test (SH-like aLRT %) [60]. Their respective domains were annotated in HMMERscan, domain trehalose-phosphatase is represented in pink. Predicted active sites are shown in green. The scale represents the number of amino acids in each protein. Databases and accession numbers are listed in Supplementary Table S1.

1.
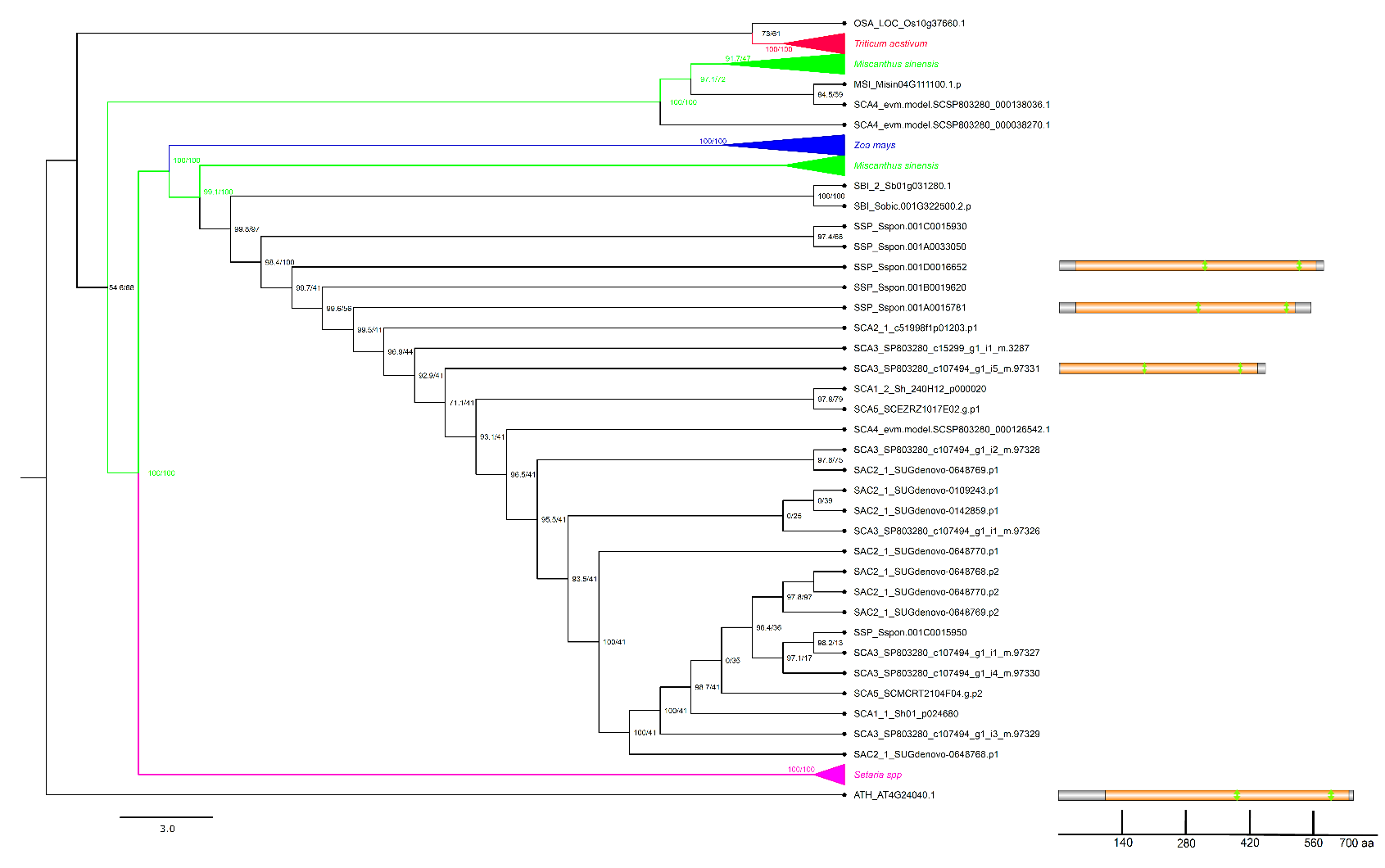
**1DYMW (OG) - TRE**

**Figure S1. l) Phylogenetic tree for the 1DYMW containing trehalase (TRE) sequences.** Phylogenetic trees were constructed from publicly protein sequences of *A. thaliana*, *Miscanthus sinensis, O. sativa*, *Setaria* spp, *S. bicolor*, and *Z. mays* datasets. The trees were built with IQ-TREE [59] using automatic evolutionary model selection and ultrafast bootstrap approximation (UFboot) [61] with branch support values improved by the approximate likelihood ratio test (SH-like aLRT %) [60]. Their respective domains were annotated in HMMERscan, domain trehalase is represented in yellow. Predicted active sites are shown in green. The scale represents the number of amino acids in each protein. Databases and accession numbers are listed in Supplementary Table S1.
