# Supplementary material for: Bioinformatic analyses to uncover genes involved in trehalose metabolism in the polyploid sugarcane
Source: Sci Rep. 2022 May 7;12:7516. doi: 10.1038/s41598-022-11508-x (PMC9079074; doi:10.1038/s41598-022-11508-x)
Supplement: Supplementary file 2 — Supplementary Information 2. [file 41598_2022_11508_MOESM2_ESM.pdf]

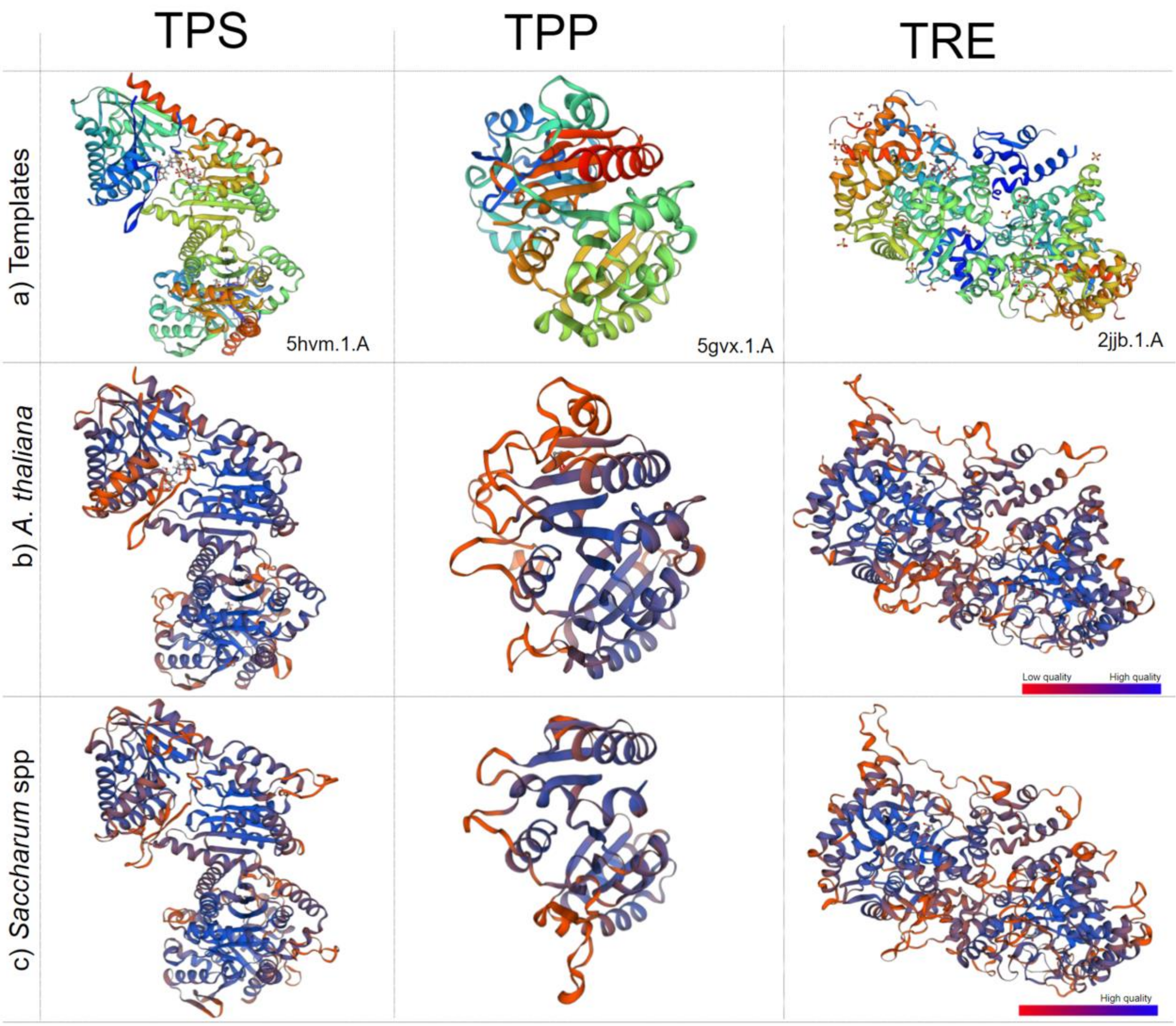

Figure S2. Three-dimensional (3D) structures of trehalose-6-phosphate synthase (TPS), trehalose-6-phosphate phosphatase (TPP), and trehalase (TRE) proteins. (a) 3D template structures of *A. fumigatus* TPS (ID model 5hvm.1.A), *M. tuberculosis* TPP (ID model 5gvx.1.A), and *E. coli* TRE (ID model 2jjb.1.A) from SWISS-MODEL (http://swissmodel.expasy.org) were used to predict protein quaternary structures of (b) *A. thaliana*, and (c) *Saccharum* spp. Sequences showed high conservation with the models indicated by colors from red (low quality) to blue (high quality). ID models, coverage, global model quality estimation (GMQE), quaternary structure quality estimate (QSQE), and identity are listed in Supplementary table S1.
