# Supplementary material for: Bioinformatic analyses to uncover genes involved in trehalose metabolism in the polyploid sugarcane
Source: Sci Rep. 2022 May 7;12:7516. doi: 10.1038/s41598-022-11508-x (PMC9079074; doi:10.1038/s41598-022-11508-x)
Supplement: Supplementary file 4 — Supplementary Information 4. [file 41598_2022_11508_MOESM4_ESM.pdf]

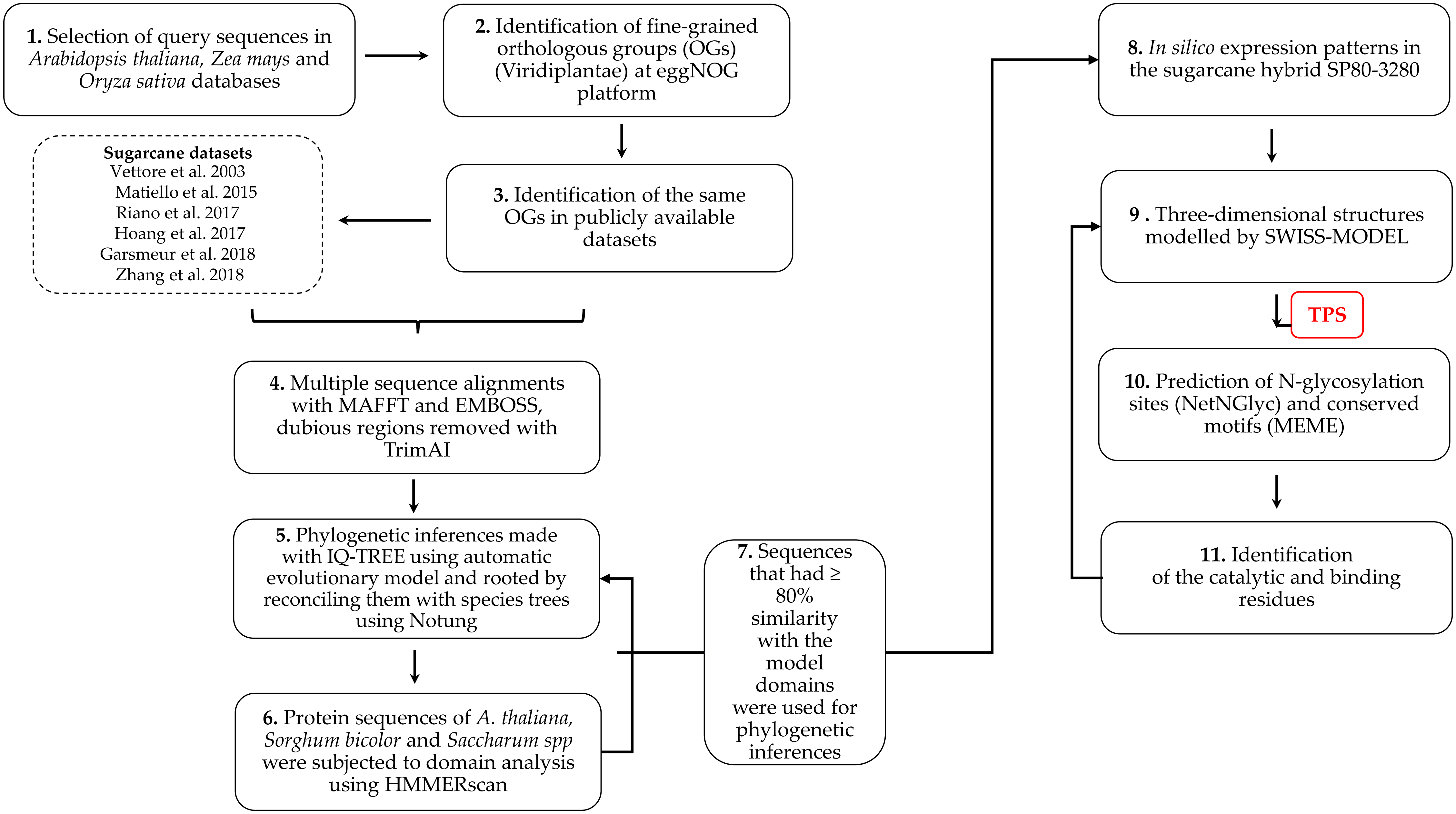

Figure S4. Schematic pipeline of the workflow for found and characterize sequences of trehalose metabolism in sugarcane.
