# Supplementary material for: Bioinformatic analyses to uncover genes involved in trehalose metabolism in the polyploid sugarcane
Source: Sci Rep. 2022 May 7;12:7516. doi: 10.1038/s41598-022-11508-x (PMC9079074; doi:10.1038/s41598-022-11508-x)
Supplement: Supplementary file 3 — Supplementary Information 3. Figure S3. Multiple protein sequence alignment of TPS Class I and II, horizontal red boxes indicate the TPS Class I sugarcane sequence with the highest expression value (SCA1_2_Sh239M11_p000040), as well as E. Coli TPS (CAA48913.1) and C. albicans Tps1 (5huu). Horizontal black boxes indicate the TPS Class II sugarcane sequence with the highest expression value (SCA3_SP803280_c117830_g1_i1_m154722) and C. albicans Tps2 (XP_721536.1). Vertical red and black boxes indicate residues involved in binding and catalysis of TPS Class I and II, respectively. All residues and possible mutations are described in Table 3. [file 41598_2022_11508_MOESM3_ESM.pdf]

CLUSTAL O(1.2.4) multiple sequence alignment

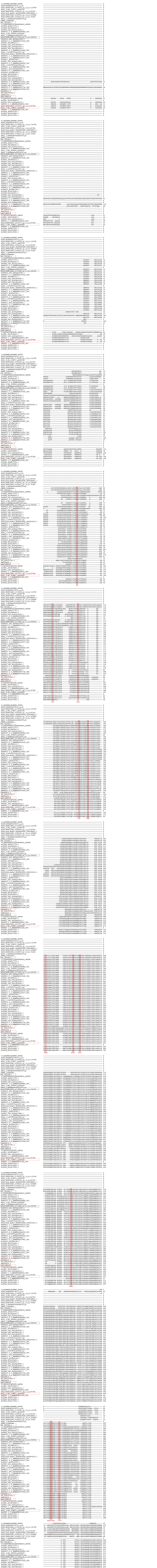

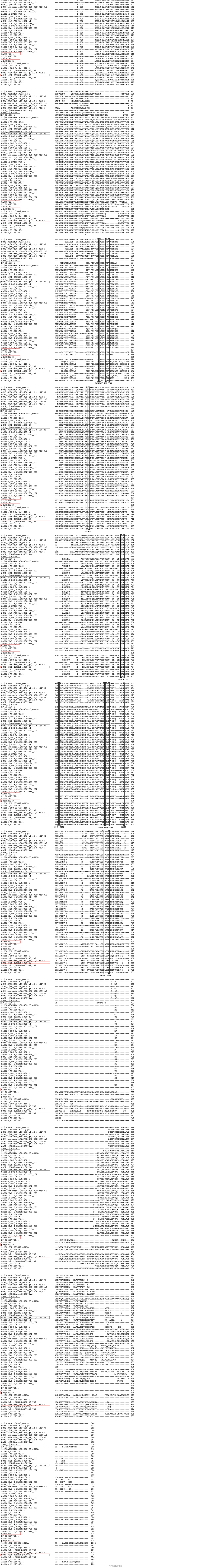

Type your text
